# Phenytoin Derivatives as Antagonists of AMPA Receptors and Voltage-Gated Sodium Channels: A Structure–Function Study

**DOI:** 10.3390/ijms27177723

**Published:** 2026-08-28

**Authors:** Arseniy S. Zhigulin, Maxim V. Nikolaev, Mikhail Y. Dron, Dmitry A. Vasilenko, Oleg I. Barygin, Denis B. Tikhonov

**Affiliations:** 1 Sechenov Institute of Evolutionary Physiology and Biochemistry, Russian Academy of Sciences, 194223 Saint Petersburg, Russia; arseniy.zhigulin@yandex.ru (A.S.Z.); fmedfstud@gmail.com (M.V.N.); neuro.mike@yahoo.com (M.Y.D.); oleg_barygin@mail.ru (O.I.B.); 2 Department of Chemistry, Lomonosov Moscow State University, 119991 Moscow, Russia; vda-ga@yandex.ru

**Keywords:** sodium channels, AMPA receptors, pore block, use-dependence, structure–function relationships

## Abstract

The development of antiepileptic drugs remains a serious challenge for both academia and industry. Ionotropic glutamate receptors and voltage-gated sodium channels are among the primary targets of antiepileptic agents. Recent studies have revealed a unique property of phenytoin: unlike other sodium-channel blockers, it inhibits calcium-impermeable AMPA receptors at micromolar concentrations. In this study, we explored the structure–activity relationships of eight phenytoin derivatives. The effects of the compounds on neuronal voltage-gated Na^+^ channels and neuronal AMPA receptor channels were examined using the patch-clamp technique. For the Na^+^ channels, we analyzed tonic block, shifts of steady-state inactivation, and frequency-dependent block. For the AMPA receptors, we investigated kinetics, agonist dependence, and trapping effects. NH groups at positions 1 and 3, the carbonyl groups at positions 2 and 4, and the phenyl group at position 5 are important, since their replacement causes a decrease in activity. Replacement of oxygen with sulfur at position 2 results in a significant increase in activity on both types of channels. For the AMPA receptor, this increase is attributable to a more stable drug–channel complex. The enhanced action on sodium channels is due to an increase in tonic block, whereas the effects on inactivated and open channels remain unchanged. These results suggest a new possibility for tuning the activities of phenytoin derivatives against both primary targets to obtain anticonvulsants with novel properties.

## 1. Introduction

There are four broad classes of anticonvulsant drug mechanisms: (1) modulation of voltage-gated sodium, calcium, and potassium channels; (2) enhancement of GABA-mediated inhibitory neurotransmission; (3) attenuation of glutamate-mediated excitatory neurotransmission; and (4) modulation of neurotransmitter release via presynaptic actions [1]. Despite efforts by both industry and academia over recent decades, many patients still do not achieve seizure control with modern medications [2]. Therefore, the search for new anticonvulsant drugs and a better understanding of their mechanisms of action remain major challenges for neuropharmacology.

The approval of perampanel, an AMPA receptor antagonist, has increased interest in AMPA receptor modulation [3,4,5,6,7]. Cationic pore blockers selectively inhibit calcium-permeable AMPAR channels (CP-AMPARs) [8], which are dominant in inhibitory neurons. Principal neurons usually express calcium-impermeable AMPA receptors (CI-AMPARs) [9]. Allosteric modulators such as perampanel affect both subtypes equally [7,10].

Given the different localization of CP-AMPARs and CI-AMPARs, agents that selectively act on CI-AMPARs appear promising as an anticonvulsive strategy, since such drugs should inhibit excitatory glutamatergic transmission while minimally affecting GABAergic transmission. Pentobarbital has shown such selectivity among known AMPA receptor modulators [11,12,13], but its multiple effects raise safety concerns and limit its clinical use. To find other ligands for CI-AMPARs, we searched for structurally related molecules with known clinical tolerability. Because pentobarbital also affects voltage-gated sodium channels [14,15,16], sodium-channel inhibitors attracted our attention.

In our previous study [17], lamotrigine, topiramate, levetiracetam, felbamate, carbamazepine, tiagabine, vigabatrin, and zonisamide did not affect AMPA receptors. Only phenytoin showed significant activity against CI-AMPARs (IC_50_ = 30 μM) and CP-AMPARs (IC_50_ = 250 μM). The affinity of phenytoin for the inactivated state of sodium channels ranges from 7 to 21 μM [18,19]. Thus, the activities of phenytoin against CI-AMPARs and sodium channels are similar, suggesting that AMPAR inhibition contributes to phenytoin’s distinct pharmacological profile compared with other sodium-channel blockers [20].

It should be noted that multitarget action is not a unique feature of phenytoin. Many clinically approved drugs act at multiple targets, and it is important to identify and validate their pharmacological profiles to develop more effective drugs [21]. For instance, levetiracetam and padsevonil act on both pre- and postsynaptic targets [22]. Another anticonvulsant, retigabine, behaves as a potassium channel opener but also acts as an allosteric positive modulator at GABA_A_ receptors [23].

**Figure 1:**
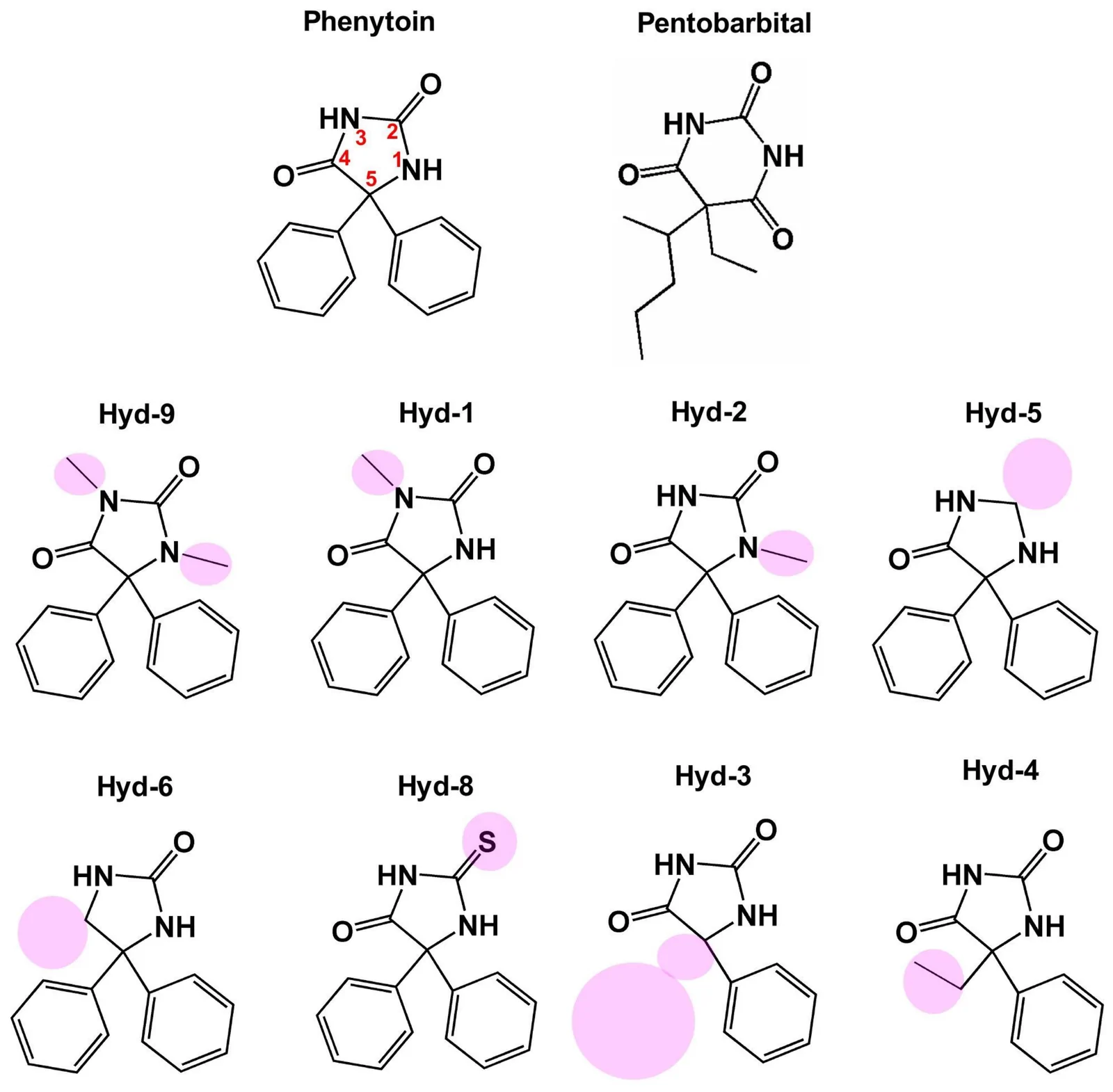
Chemical structures of pentobarbital, phenytoin, and phenytoin derivatives. Modification areas of the phenytoin molecule are shown in color. The position numbers in the hydantoin ring are indicated.

The structural similarity between phenytoin and pentobarbital (Figure 1) provides a rationale for their similar actions on sodium channels and CI-AMPARs. Both drugs contain a heterocyclic ring with hydrogen-bond donors and acceptors, as well as hydrophobic/aromatic groups. The details of pentobarbital’s and phenytoin’s actions on AMPARs are similar [17]. A systematic analysis of structure–function relationships is needed to design novel antiepileptic drugs with different activity ratios against the targets. In this study, we designed and tested eight phenytoin derivatives to identify key structural determinants of their action on both AMPA receptors and sodium channels. All examined moieties proved important for activity. A compound with sulfur replacing oxygen at position 2 of the hydantoin ring showed improved activity against both sodium channels and AMPA receptors.

## 2. Results

### 2.1. Design of Phenytoin Derivatives

To systematically address structure–function relationships of phenytoin derivatives, we introduced several modifications to the phenytoin molecule (Figure 1). The five-membered heterocyclic ring (hydantoin) contains nitrogens at positions 1 and 3 and carbonyl groups (C=O) at positions 2 and 4. Two bulky, lipophilic phenyl groups are attached at position 5. We examined how methylation of the NH groups at positions 1 and 3 affects activity. Compounds Hyd-1, Hyd-2, and Hyd-9 have increased steric bulk and lack one or two hydrogen-bond donors. Compounds Hyd-5 and Hyd-6 lack the carbonyl groups at positions 2 and 4 and therefore cannot act as hydrogen-bond acceptors at those positions. Hyd-8 tests an oxygen-to-sulfur substitution at position 2 (5,5-diphenyl-2-thiohydantoin). Compounds Hyd-3 and Hyd-4 have modifications of the diphenyl moiety: in Hyd-3, one phenyl ring was removed, and in Hyd-4, it was replaced by an ethyl group. Together, these modifications allow us to evaluate the influence of local structural changes on the activity against AMPA receptors and sodium channels.

### 2.2. Screening for Activity Against AMPA Receptors Among Phenytoin Derivatives

**Figure 2:**
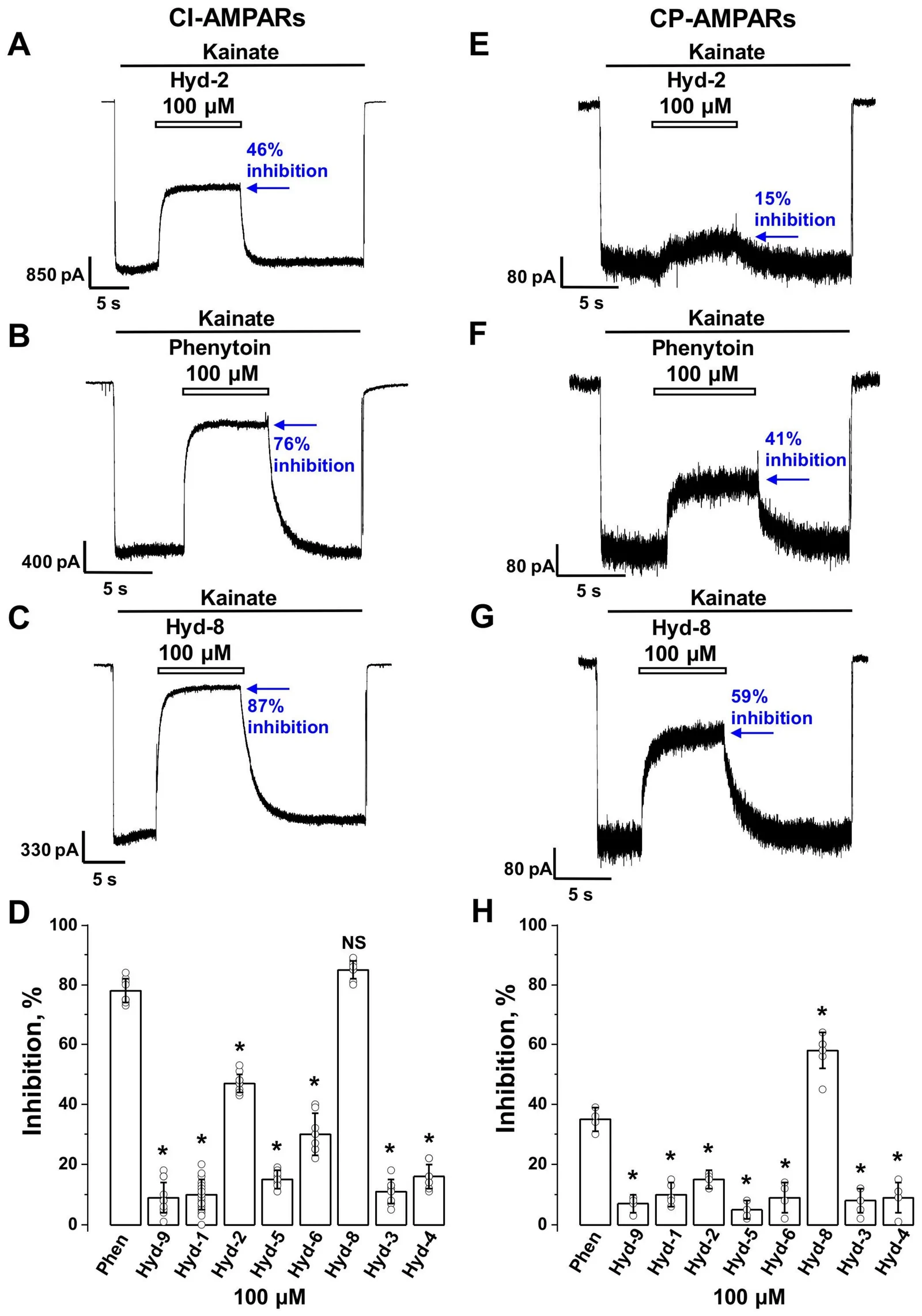
Screening for activity against AMPA receptors among phenytoin derivatives. (**A**–**C**). Representative examples of CI-AMPAR inhibition by 100 μM of Hyd-2 (**A**), phenytoin (**B**), and Hyd-8 (**C**). (**D**) Summary of the data on CI-AMPARs. All compounds, except Hyd-8, were less active than phenytoin. *-*p* < 10^−6^, ANOVA with Tukey post hoc test. (**E**–**G**). Representative examples of CP-AMPAR inhibition by 100 μM of Hyd-2 (**E**), phenytoin (**F**), and Hyd-8 (**G**). (**H**) Summary of the data on CP-AMPARs. Only Hyd-8 was more potent than phenytoin, while other phenytoin derivatives were less effective. *-*p* < 10^−6^, NS–not significant, ANOVA with Tukey post hoc test. In (**D**,**H**) circle symbols show individual values.

We initially tested whether phenytoin derivatives at 100 μM affect kainate-induced currents at a holding potential of −80 mV. None of the compounds at this concentration caused significant changes in holding current or input resistance. Application of 100 μM kainate to isolated hippocampal CA1 pyramidal neurons and giant striatal interneurons elicited weak or non-desensitizing inward currents. Phenytoin and its derivatives produced rapid and fully reversible inhibition of kainate currents mediated by both CP- and CI-AMPARs (see Figure 2 and Table 1).

Removal of both proton donors in Hyd-9 resulted in a dramatic loss of activity, bringing it to levels similar to previously tested carbamazepine (Table 1) and other inactive compounds [17]. However, individual NH substitutions had different effects. Hyd-1 showed almost the same weak activity as Hyd-9 (*p* > 0.05, one-way ANOVA with Tukey’s post hoc test, *n* = 11–17), whereas Hyd-2 displayed intermediate activity between phenytoin and Hyd-1/Hyd-9 (*p* < 10^−6^ for Hyd-2 vs. phenytoin, *n* = 8–12; *p* < 10^−6^ for Hyd-2 vs. Hyd-1; *p* < 10^−6^ for Hyd-2 vs. Hyd-9; one-way ANOVA with Tukey’s post hoc test). These results strongly suggest that both hydrogen-bond donors contribute to activity, with the donor at position 3 appearing to be critical. Most likely, methylation at this position in Hyd-1 prevents productive binding at the specific site, whereas methylation at position 1 (Hyd-2) only reduces affinity.

Compounds Hyd-5 and Hyd-6 both showed a strong reduction of activity compared with phenytoin (*p* < 10^−6^ for Hyd-5 vs. phenytoin, *n* = 8–9; *p* < 10^−5^ for Hyd-6 vs. phenytoin, *n* = 8; one-way ANOVA with Tukey’s post hoc test), indicating the importance of carbonyl groups as hydrogen-bond acceptors for productive binding. By contrast, the sulfur-containing Hyd-8 was highly potent and similar to phenytoin (*p* = 0.06 for Hyd-8 vs. phenytoin, *n* = 8; one-way ANOVA with Tukey’s post hoc test). The weak activity of Hyd-3 and Hyd-4 (*p* < 10^−6^ for Hyd-3 vs. phenytoin, *n* = 8; *p* < 10^−6^ for Hyd-4 vs. phenytoin, *n* = 8; one-way ANOVA with Tukey’s post hoc test) demonstrates that the diphenyl moiety of phenytoin is also important for activity against AMPA receptors.

In all cases, inhibition of CP-AMPARs was weaker than inhibition of CI-AMPARs. However, the structure–activity relationships were similar: phenytoin and Hyd-8 were more active than the other derivatives against both AMPAR subtypes.

**Figure 3:**
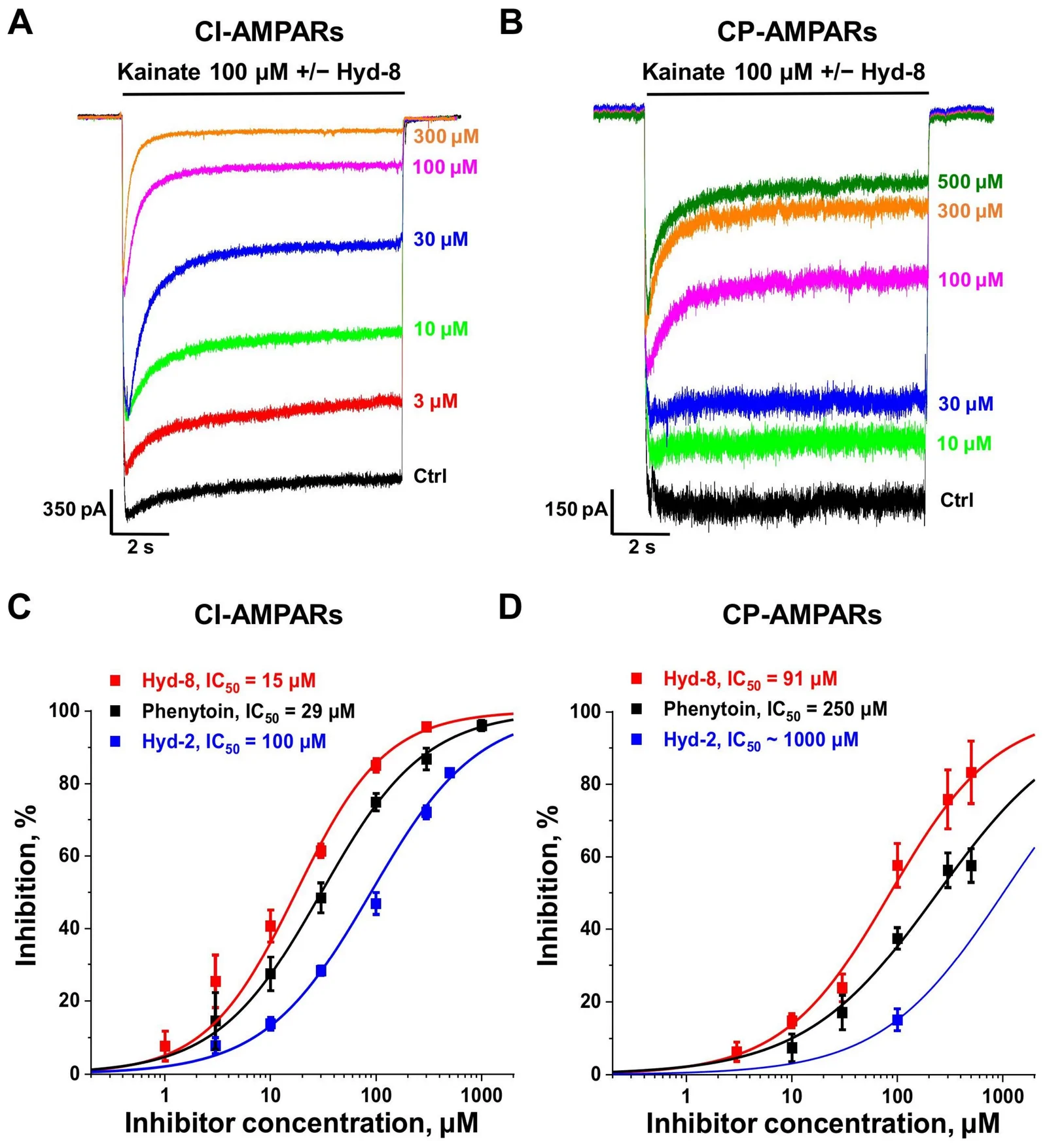
Concentration dependence of Hyd-8 and Hyd-2 in comparison with phenytoin on CI- and CP-AMPARs. A, B Representative examples of CI- (**A**) and CP-AMPAR (**B**) inhibition by different concentrations of Hyd-8. (**C**,**D**) Summary of the data on CI- (**C**) and CP-AMPARs (**D**).

Although the simple single-concentration screen discriminates between active and inactive compounds, it does not provide precise comparisons within the active group. ANOVA did not reveal statistically significant differences between the effects of Hyd-8 and phenytoin on CI-AMPARs. On the other hand, Hyd-2 was significantly less active than phenytoin and Hyd-8, but more active than the other derivatives. Therefore, we analyzed the full concentration–response relationships for phenytoin, Hyd-2, and Hyd-8; the results are shown in Figure 3. In these experiments, kainate was applied simultaneously with the blocking drugs. The development of the block is seen as the response decay to a steady-state level that was measured and further analyzed. Since the rate of response development includes a component which is proportional to concentration, the response decay accelerated with drug concentration.

Fitting the concentration-dependence data on the steady-state level of inhibition for CI-AMPARs with the Hill equation gave IC_50_ values of 29 ± 7 (*n* ≥ 4 for each concentration) μM for phenytoin, 100 ± 8 μM (*n* ≥ 4) for Hyd-2, and 15 ± 3 μM (*n* ≥ 6) for Hyd-8. Thus, Hyd-8 is approximately twofold more potent than phenytoin at inhibiting CI-AMPARs. For CP-AMPARs, the IC_50_ values for phenytoin and Hyd-8 were 250 ± 60 μM and 91 ± 26 μM (*n* ≥ 4), respectively. Hill coefficients were close to 1 in all, suggesting the absence of cooperativity effects. Note the similar potency ratios between phenytoin and Hyd-8 for both AMPAR subtypes.

### 2.3. Mechanisms of Hyd-2 and Hyd-8 Action on CI-AMPARs: Comparison with Phenytoin

Chemical modification can affect overall drug activity in several ways. Besides altering complex stability, it can change accessibility of the binding site and the position and orientation of the drug molecule within the site. Changes in these characteristics can in turn affect mechanisms of action such as kinetics, agonist dependence, and use dependence. These features of inhibition are critical for drug action in physiological and pathological conditions. Therefore, we systematically compared the actions of phenytoin, Hyd-2, and Hyd-8 on their primary target, CI-AMPARs.

**Figure 4:**
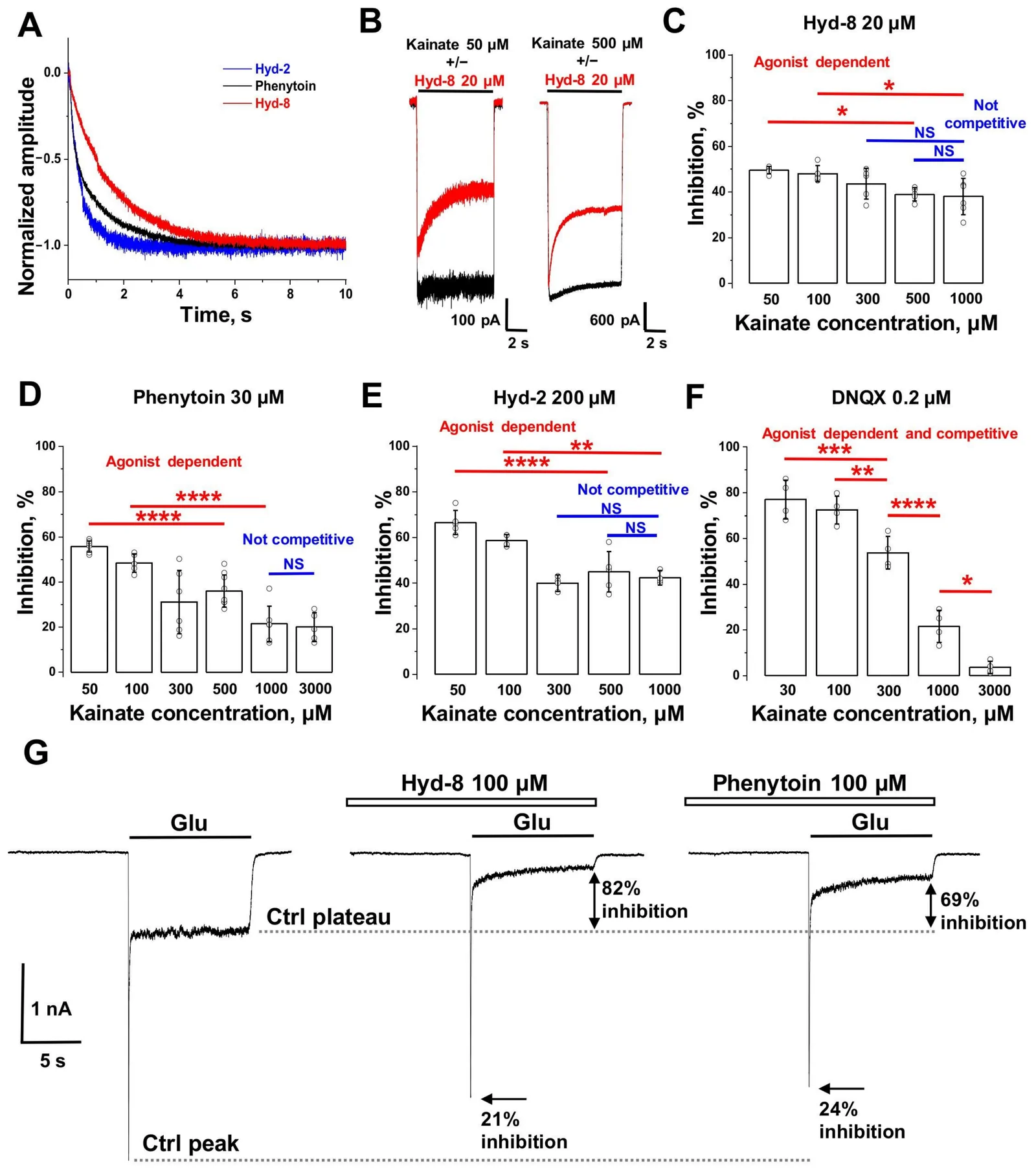
Analysis of recovery kinetics and agonist dependence for Hyd-8, phenytoin, and Hyd-2 on CI-AMPARs. (**A**). Example of recovery kinetics for Hyd-2, phenytoin, and Hyd-8, demonstrating that the less-active compound (Hyd-2) has the fastest recovery, while more-active Hyd-8 has the slowest one. B-F. Agonist dependence of CI-AMPAR inhibition by 20 μM Hyd-8 ((**B**)—examples at kainate 50 vs. 500 μM; (**C**)—summary of the data), 30 μM phenytoin (**D**), 200 μM Hyd-2 (**E**) and 0.2 μM DNQX (**F**). Inhibition by Hyd-8, phenytoin and Hyd-2 is agonist-dependent only at lower kainate concentrations, suggesting a noncompetitive action. In contrast, inhibition by the competitive AMPAR antagonist DNQX is agonist-dependent across the whole range of kainate concentrations. * *p* < 0.05, ** *p* < 0.01, *** *p* < 0.005, **** *p* < 0.0001, NS—not significant, ANOVA with Tukey’s post hoc test. (**G**) Representative recording of the glutamate-induced response of CI-AMPARs under control conditions and in the presence of Hyd-8 or phenytoin. Deep inhibition of the steady-state response is accompanied by weak inhibition of the peak response, suggesting some binding to closed channels.

First, we analyzed the kinetics of action using the experimental protocol shown in Figure 2A–C. The rate of inhibition development is comparable with the solution-exchange time (~200 ms). Recovery is slower because it is determined by the dissociation (off-rate) constant and can be precisely estimated from the experiments, as shown in Figure 4A. In that panel, current recordings are scaled so that equilibrium inhibition corresponds to zero and the recovery level coincides with −1 (control response). Hyd-2 demonstrated the fastest recovery kinetics and Hyd-8 the slowest. Monoexponential fits yielded recovery rate constants of 2.2 ± 0.4 s^−1^ (*n* = 11), 0.9 ± 0.2 s^−1^ (*n* = 6), and 0.6 ± 0.1 s^−1^ (*n* = 8) for Hyd-2, phenytoin, and Hyd-8, respectively. The ratios of these values correlate with the ratios of IC_50_ values: 0.5:1:3.4 (IC_50_s) and 0.7:1:2.5 (recovery constants) for Hyd-2, phenytoin, and Hyd-8. This indicates that differences in activity are mainly due to differences in recovery rate, which reflect drug–channel complex stability.

A previous study [17] showed that phenytoin action depends on agonist concentration: inhibition weakens at high kainate concentrations, although the action is not competitive. We reproduced these results and made analogous measurements for Hyd-2 and Hyd-8. The inhibitory effect of Hyd-8 (20 μM) was reduced at higher kainate concentrations (Figure 4B,C); the same was true for phenytoin (30 μM, Figure 4D) and Hyd-2 (200 μM, Figure 4E). The main feature of all three compounds is that agonist dependence appears at lower agonist concentrations, whereas at higher concentrations the inhibitory effect stabilizes, implying that phenytoin and its derivatives do not compete with kainate for the binding site. By contrast, the competitive AMPAR antagonist DNQX (0.2 μM) showed a drastic change in activity across the full range of kainate concentrations (Figure 4F).

Although kainate is suitable as an AMPAR activator for in vitro experiments, activation by glutamate is more tightly related to physiological situation. Therefore, we tested action of continuously applied phenytoin and Hyd-8 on the CI-AMPAR responses to 1 mM glutamate. D-AP5 (100 μM) was present to ensure the absence of NMDAR-mediated component of the response. Hyd-8 or phenytoin in 100 μM concentration was applied 10 s before and during CI-AMPAR activation. The inhibitory effects on the steady-state response were 80 ± 3% (*n* = 5) and 71 ± 2% (*n* = 5) for Hyd-8 and phenytoin, respectively. These values are similar to those obtained for kainate-induced currents (see above). Moreover, both compounds slightly inhibited the peak component of the response, suggesting binding to closed channels (Figure 4G).

**Figure 5:**
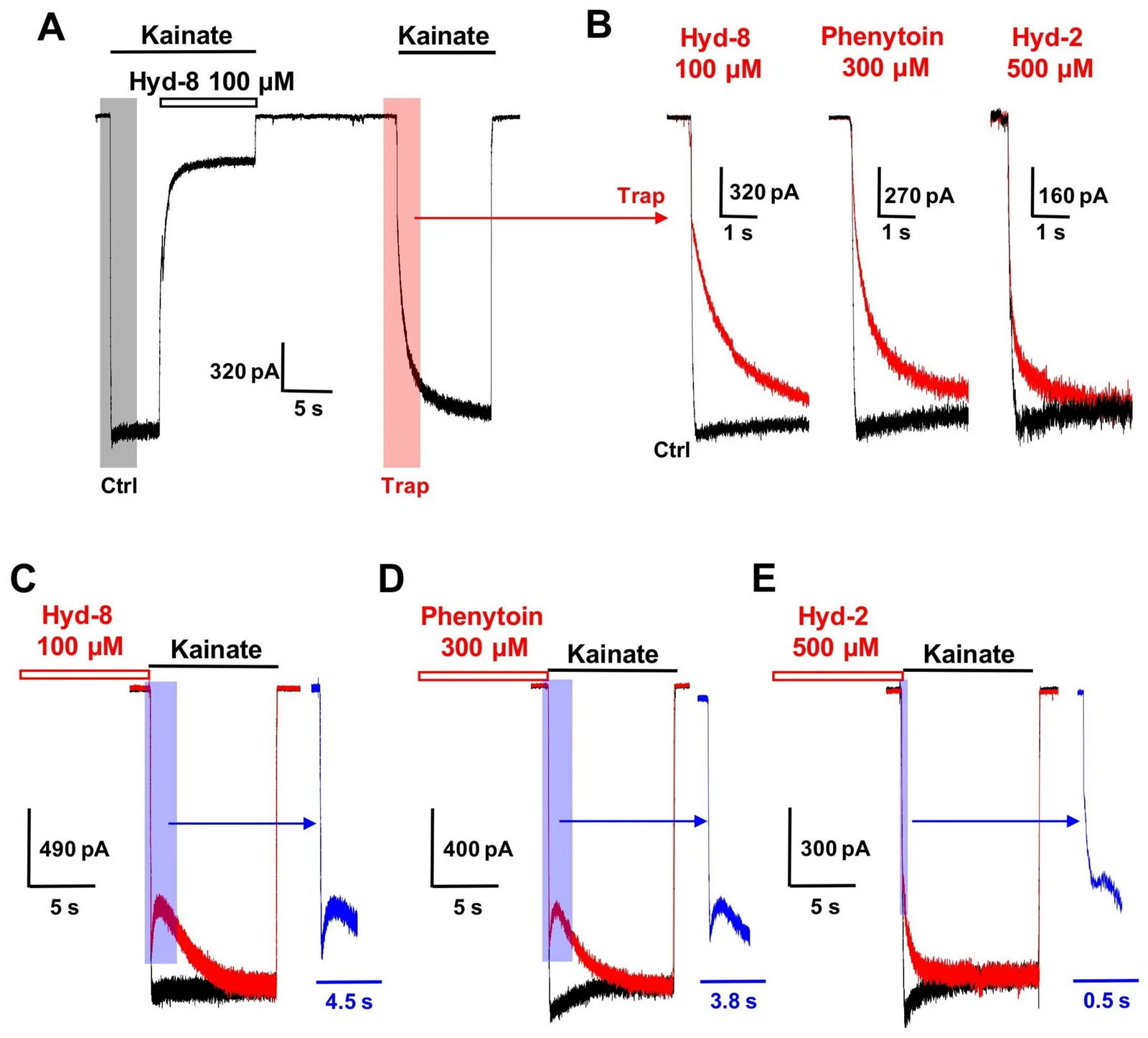
Mechanisms of inhibition of open and closed CI-AMPAR channels by phenytoin, Hyd-8, and Hyd-2. A, B. All compounds demonstrate a trapping effect. (**A**) A representative example of the protocol used, Hyd-8 (100 μM), is shown. The control response is colored in black, while the testing response, whose inhibition indicates trapping, is shown in red. (**B**) Representative comparison of control and testing responses for Hyd-8 (100 μM), phenytoin (300 μM), and Hyd-2 (500 μM). (**C**–**E**) All compounds demonstrate binding to closed channels. Hyd-8 (100 μM, (**C**)), phenytoin (300 μM, (**D**)), and Hyd-2 (500 μM, (**Ε**)) were applied before receptor activation. The control kainate response is shown in black, while the testing response after compound preapplication is shown in red. The blue trace shows the kinetics of the testing response inhibition in detail.

An important property of phenytoin action on CI-AMPARs is the trapping effect. Trapping means that drug binding does not prevent channel closure, but the bound drug cannot leave the channel until it reopens. As a result, recovery in the absence of agonist is much slower than in its presence. To compare trapping by phenytoin, Hyd-2, and Hyd-8, we used concentrations that produced similar levels of block: 100 μM Hyd-8, 300 μM phenytoin, and 500 μM Hyd-2 (~80% inhibition). The protocol in Figure 5A includes: (1) recording the control response; (2) inducing deep block by drug, then simultaneously removing drug and agonist; and (3) testing agonist application after a delay sufficient for full recovery in the presence of agonist. Trapping was estimated by comparing control and test responses to agonist. The test response shows two phases: a fast component reflecting the fraction of unblocked channels, and a slow component reflecting unbinding of blocker molecules that remained bound after agonist removal. A visible inflection in the test response was detected for all three compounds, indicating that they all exhibit trapping.

Although open-channel block with trapping is the main mechanism of CI-AMPAR inhibition by phenytoin, this drug is uncharged at physiological pH (pKa = 8.33, see https://pubchem.ncbi.nlm.nih.gov) and can enter the channel via the membrane lipid phase [24]. This mechanism is analogous to tonic block of sodium channels, where drugs access the binding site from the membrane through the interface between pore-lining segments [25]. Evidence for this membrane-to-channel pathway of phenytoin, Hyd-2, and Hyd-8 on CI-AMPARs is illustrated in Figure 5C–E. The drugs were applied in the absence of kainate and removed just before channel activation. The initial response component was already inhibited, indicating that some drug molecules entered the channel before activation. Moreover, for all three compounds, inhibition continued to develop (Figure 5C–E, blue traces) because a fraction of drug molecules remained in the membrane even after washout from the external solution. Thus, complex shape of response reflects two effects: initial block by the drug accumulated in the membrane and slow unbinding after receptor activation.

To verify that this effect requires the deprotonated state of the drugs, we compared binding to closed channels at pH 7.4 vs. pH 9.5 (Figure S19). At pH 9.5, the compounds are predominantly negatively charged and should have poor permeability through the lipid layer. All three compounds were significantly less active at pH 9.5, supporting the conclusion that phenytoin and its derivatives access CI-AMPARs via a membrane-to-channel path.

In summary, Hyd-2 and Hyd-8 share the same CI-AMPAR inhibition mechanism as phenytoin; they differ from the parent drug primarily in potency, which is reflected in corresponding differences in the recovery rate constants.

### 2.4. Screening for Activity Against Voltage-Gated Sodium Channels Among Phenytoin Derivatives

**Figure 6:**
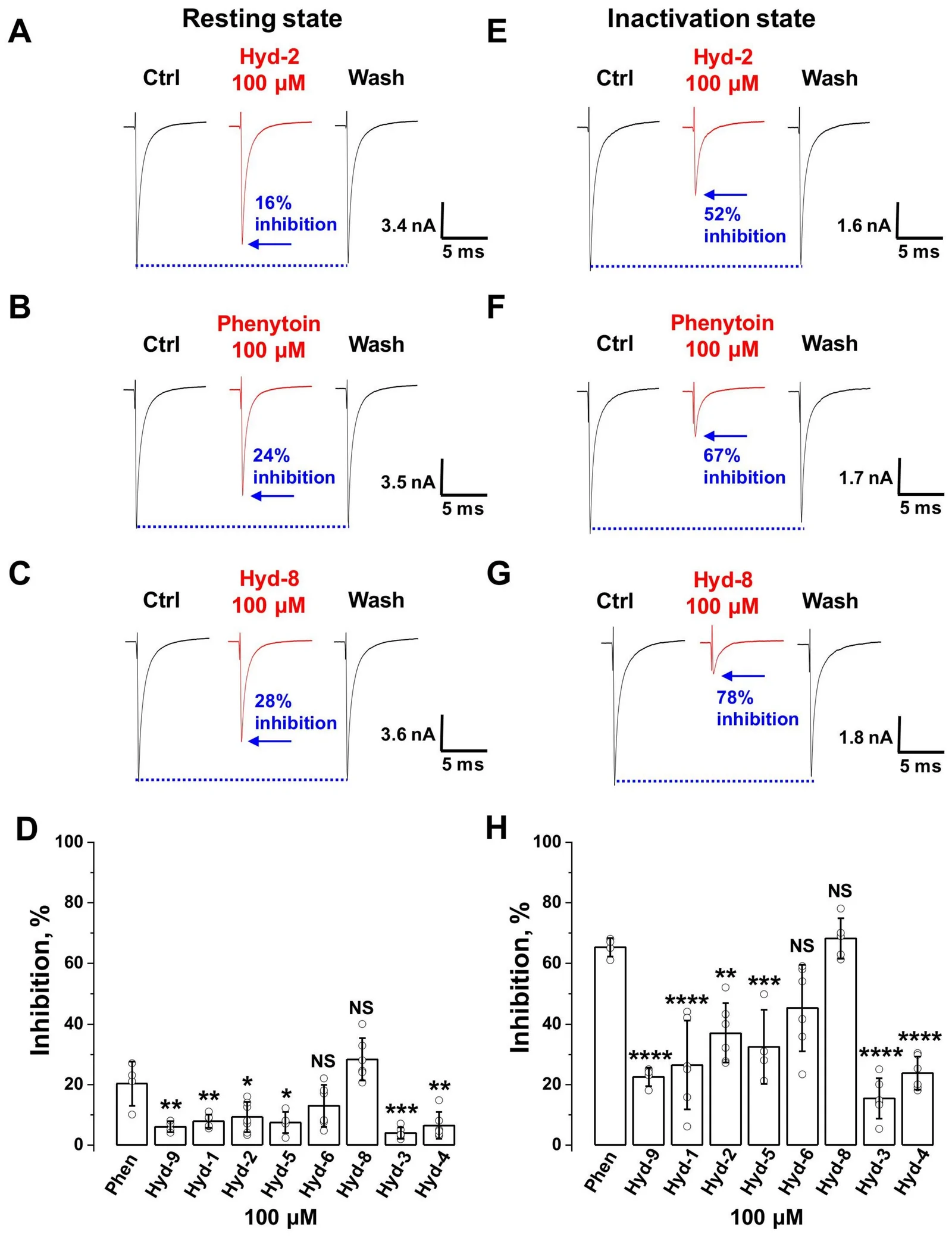
Screening for activity against VGSCs among phenytoin derivatives. A-C Representative examples of tonic block produced by 100 μM Hyd-2 (**A**), phenytoin (**B**), and Hyd-8 (**C**). (**D**) Summary of the data on tonic block. All compounds except Hyd-6 and Hyd-8 were less active than phenytoin. * *p* < 0.05, ** *p* < 0.01, *** *p* < 0.001, ANOVA with Tukey’s post hoc test. (**E**–**G**) Representative examples of inactivated VGSCs inhibition by 100 μM Hyd-2 (**E**), phenytoin (**F**), and Hyd-8 (**G**). (**H**) Summary of the data on inhibition in the inactivated state. All compounds except Hyd-6 and Hyd-8 were less active than phenytoin. ** *p* < 0.01, *** *p* < 0.001, **** *p* < 0.0001, NS—not significant, ANOVA with Tukey’s post hoc test.

Because the classical neuronal targets of phenytoin are voltage-gated sodium channels (VGSCs), all compounds were tested for their ability to block voltage-activated sodium currents in isolated CA1 pyramidal neurons. To compare with the effects on AMPARs, a concentration of 100 μM was used. A commonly used protocol to assess tonic block activates channels by a voltage step from −110 mV to 0 mV for 40 ms with 5 s intervals. Drugs were applied extracellularly after control recordings and washed out after equilibration of the effect. A typical experiment is presented in Figure 6A–C; results are summarized in Figure 6D and Table 2. Tonic block generally developed during a 1 min application and recovery required ~1.5 min of washout.

Only phenytoin and Hyd-8 produced >20% inhibition. Thus, tonic block of VGSCs by phenytoin and its derivatives resembles the inhibition of CP-AMPARs and is weaker than inhibition of CI-AMPARs. Hyd-8 and Hyd-6 produced tonic block similar to that produced by phenytoin (*p* = 0.36 for Hyd-6 vs. phenytoin, *n* = 4–6; *p* = 0.24 for Hyd-8 vs. phenytoin, *n* = 4–6; one-way ANOVA with Tukey’s post hoc test), whereas the other compounds were less active (Figure 6D). However, tonic block of VGSCs by local anesthetics and anticonvulsants, which reflects interaction with predominantly closed channels, is often subtle. Functionally important effects result from stronger block of inactivated VGSCs. This distinction is a key element of the “modulated receptor” hypothesis [26].

To induce steady-state inactivation, we used a pre-conditioning holding potential of −80 mV for 1 s. Under these conditions, the response to the step to 0 mV was approximately twofold smaller. Representative recordings are shown in Figure 6E–G. When steady-state inactivation was promoted, the inhibitory effect of all compounds markedly increased (Figure 6H, Table 2). Phenytoin and Hyd-8 were the most active, producing >60% inhibition. There was no significant difference between phenytoin and either Hyd-8 or Hyd-6 (*p* = 0.99 for Hyd-8 vs. phenytoin, *n* = 4–6; *p* = 0.07 for Hyd-6 vs. phenytoin, *n* = 4–6; one-way ANOVA with Tukey’s post hoc test). However, Hyd-6 and Hyd-8 differed significantly (*p* = 0.01, *n* = 5–6; one-way ANOVA with Tukey’s post hoc test). Other derivatives were significantly less active than phenytoin (Figure 6H). Hyd-3 showed the lowest activity, with only ~15% inhibition. Notably, structural modifications of phenytoin affected activity against VGSCs to a lesser extent than they affected activity against CI-AMPARs (see Table 1 and Table 2).

### 2.5. Comparison of Phenytoin and Hyd-8 Action on Voltage-Gated Sodium Channels

**Figure 7:**
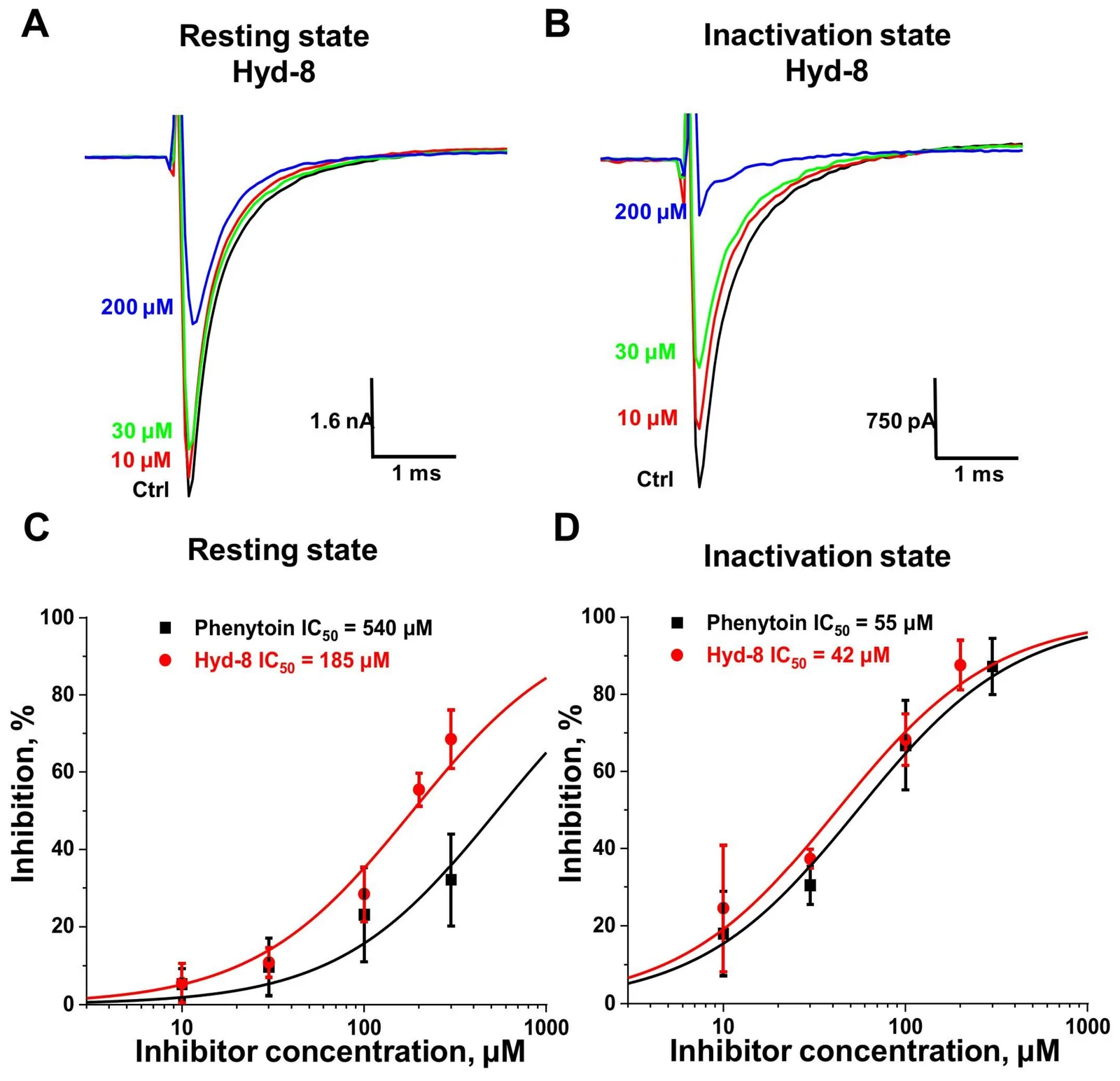
Concentration dependence of Hyd-8 and phenytoin for the resting and inactivated states of VGSCs. (**A**,**B**) Representative examples of inhibition of VGSCs in the resting (**A**) and inactivated (**B**) states by different concentrations of Hyd-8. (**C**,**D**) Summary of the data on the resting (**C**) and inactivated states (**D**).

To analyze the differences between phenytoin and Hyd-8 in detail, we first estimated the concentration dependence of their actions in the two experimental protocols described above. Figure 7 shows that the main difference between the drugs is that Hyd-8 is significantly more active in the tonic-block protocol. The IC_50_ value of phenytoin (540 ± 80 μM, *n* ≥ 4 for each concentration) is almost three times higher than that of Hyd-8 (185 ± 14 μM, *n* ≥ 4). In the protocol with stimulated inactivation, the IC_50_ values are close to each other (55 ± 6 μM and 42 ± 5 μM, respectively). Hill coefficients were ~1 in all cases. Thus, oxygen-to-sulfur replacement affects interaction with resting but not with inactivated channels.

**Figure 8:**
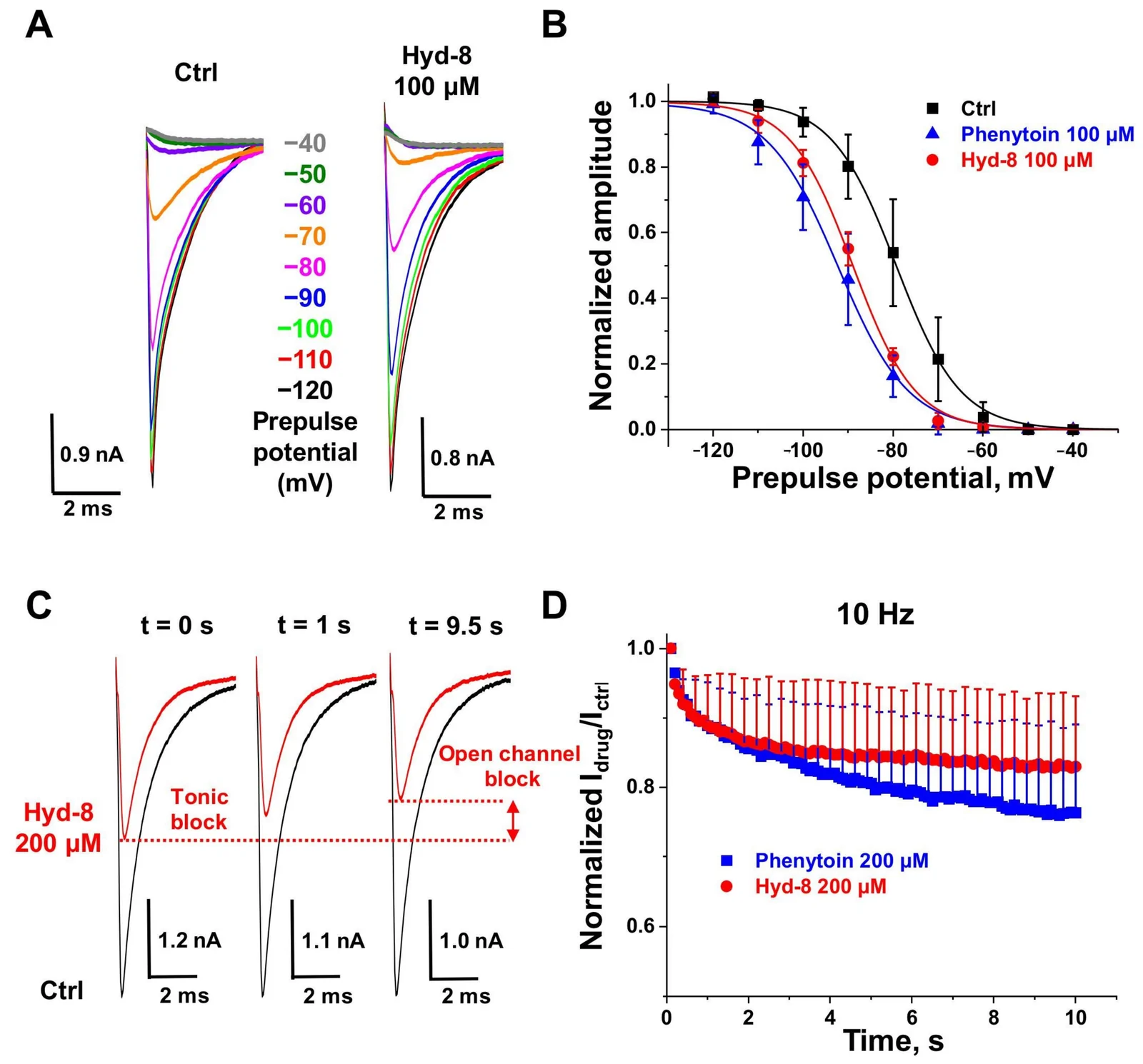
Mechanisms of VGSC inhibition by Hyd-8 and phenytoin. (**A**,**B**) Influence of Hyd-8 and phenytoin (100 μM) on the steady-state inactivation curve. Both compounds shift the inactivation curve to hyperpolarized potentials. (**A**) Representative examples of sodium currents activated by a voltage step to 0 mV from different prepulse potentials (from −120 to −40 mV) under control conditions (**left**) and in presence of Hyd-8 (**right**). (**B**) Inactivation curves under control conditions and in the presence of Hyd-8 and phenytoin. (**C**,**D**) Study of open-channel block. VGSCs were activated using 10 Hz trains of voltage steps from −110 to 0 mV for 10 s under control conditions and after tonic block achievement by Hyd-8 or phenytoin (200 μM). (**C**) Representative examples of Hyd-8 action at t = 0 s (tonic block), t = 1 s and t = 9.5 s. The inhibitory effect increases with time, indicating additional block of open channels. (**D**) Summary of normalized effects for Hyd-8 and phenytoin. The level of open-channel block is similar for both compounds and does not exceed 20%.

Moreover, since the conditioning depolarization causes only partial inactivation, IC_50_ values obtained in the inactivation protocol are expected to be higher than the true K_i_ values for inactivated channels. For a reliable estimation of these parameters, we systematically studied the relationship between the prepulse potential that induces steady-state inactivation and the actions of phenytoin and Hyd-8. In this series, the prepulse voltage was varied under control conditions and in the presence of 100 μM phenytoin or Hyd-8. The results are shown in Figure 8. Both compounds have a similar effect on the steady-state inactivation curve. They cause negative shifts of V_1/2_ from −79 ± 5 mV under control conditions to −94 ± 4 mV and −87 ± 2 mV in the presence of phenytoin and Hyd-8, respectively. Next, using Equation (2) (see Methods), we calculated K_i_ values for phenytoin and Hyd-8, which appeared to be ~20 μM for both compounds (*n* = 4, *p* = 0.09, unpaired t-test with Welch correction). These K_i_ values are indeed lower than the IC_50_ values for the inactivated state obtained above. Moreover, the Ki value obtained for phenytoin is in good agreement with previous results [27]. Thus, the affinity of Hyd-8 for inactivated VGSCs is the same as that of phenytoin, and the difference in activity of the two drugs is due only to different interactions with closed VGSCs.

The fast transient nature of VGSC responses prevents direct estimation of drug action on open channels. Indirectly, such action is described as frequency-dependent block. Instead of the 0.2 Hz activation used for estimation of tonic block, VGSCs were activated by trains of 10 Hz voltage steps for 10 s. In this protocol, the total time channels spend in the open state is increased. If a drug has higher affinity for open channels than for closed channels, inhibition increases during the train. First, we applied the protocol in the absence of compounds; the difference between the last and the first pulse amplitudes did not exceed 10%. After response recovery, we applied phenytoin or Hyd-8 at a concentration of 200 μM and achieved equilibrium tonic block at 0.2 Hz. Then we activated VGSCs at 10 Hz again and estimated I_drug_/I_ctrl_ for each pulse. Representative examples of Hyd-8 action at t = 0 s (first pulse), 1 s, and 9.5 s are presented in Figure 8C. The I_drug_/I_ctrl_ ratio decreased with time for both compounds, suggesting that both phenytoin and Hyd-8 produce additional open-channel block. The data were normalized for the tonic-block effect (I_drug_/I_ctrl_ at t = 0 s); the results are shown in Figure 8D. To estimate open-channel block, we fitted the data with a single-exponential function and obtained plateau values for the normalized I_drug_/I_ctr_l ratios, which were not significantly different for phenytoin and Hyd-8 (0.75 ± 0.13 vs. 0.83 ± 0.10, respectively; *n* = 4, *p* = 0.38, unpaired t-test). Note that the result for phenytoin is in good agreement with previously published data [27]. Thus, phenytoin and Hyd-8 equally inhibit open VGSCs with relatively low affinity compared with their affinities for closed and inactivated states.

## 3. Discussion

Interest in AMPA receptors as targets for anticonvulsant action has increased significantly following the discovery of the antiepileptic effect of perampanel. In contrast to cationic pore blockers, perampanel inhibits CP-AMPARs and CI-AMPARs equally [7], while cationic pore blockers selectively inhibit CP-AMPARs [8,28]. Among known pharmacological agents, only pentobarbital preferentially blocks CI-AMPARs [11,12,13], but the use of pentobarbital is strongly limited by undesirable side effects. Pentobarbital is also known to affect voltage-gated sodium channels [29]. Therefore, in the search for novel blockers of CI-AMPARs, we screened anticonvulsants that act as sodium-channel blockers [17]. We found that the classical anticonvulsant phenytoin inhibits AMPAR channels at micromolar concentrations. Key characteristics of phenytoin action on AMPARs coincide with those of pentobarbital.

The preferential action of phenytoin on CI-AMPARs versus CP-AMPARs results from the difference at the selectivity filter (Q/R site). This site contains Gln and Arg residues in CP-AMPARs and CI-AMPARs, respectively [9]. Neutral Gln favors calcium permeability and block by cationic ligands [30]. Positively charged Arg prevents calcium permeation and favors interactions with neutral ligands like phenytoin and its derivatives, as well as pentobarbital [11,17].

Selective inhibition of CI-AMPARs by phenytoin and its clinical tolerability inspired us to use phenytoin as a structural template to search for new drugs with combined activity against CI-AMPARs and voltage-gated sodium channels. In the present study, we tested eight compounds with modifications introduced into different parts of the phenytoin molecule. Most modifications resulted in decreased activity, suggesting that the phenytoin scaffold is well tuned for specific binding to both targets. However, oxygen-to-sulfur replacement at position 2 (Hyd-8) caused a significant increase in activity against both targets. Sulfur is larger than oxygen; its electron cloud is more diffuse, and its orbitals are less directional. The thiocarbonyl group C=S is more effective than the carbonyl C=O in noncovalent interactions in which high polarizability is important rather than electrostatic attraction, for example, in van der Waals interactions. In classical strong hydrogen bonds, oxygen is superior to sulfur because the electrostatic contribution is crucial. Conversely, the C=S group can accept weakly polarized protons, which a rigid oxygen atom does not. The difference between Hyd-8 and phenytoin suggests that the H-bond acceptor at position 2 does not participate in classical hydrogen bonds or strong electrostatic interactions.

We further investigated the actions of phenytoin and Hyd-8 in more detail because chemical modifications can affect drug activity in multiple ways, and mechanisms of action on ion channels often determine effects in physiological and pathological conditions. For example, the kinetics and mechanism of action determine the effectiveness and tolerability of the NMDAR antagonist memantine [31] and the AMPAR antagonist perampanel [5,32,33]. We found that Hyd-8 acts on AMPA receptors and voltage-gated sodium channels in the same manner as phenytoin. On AMPARs, both drugs demonstrate pore block with trapping in the closed state. Besides binding to the open channel, they can access the pore site via the membrane (membrane-to-channel mechanism). The membrane-to-channel mechanism implies accumulation of the blocker in the lipid bilayer while channels are closed; after channel opening the drug moves from the membrane site into the channel, resulting in pore block. Compared with classical open-channel blockers, drugs that can reach the channel through the membrane are more promising for inhibiting the fast component of excitation. The membrane-to-channel mechanism was previously described for AMPAR block by fluoxetine [34]. Later it was shown that memantine, MK-801, phencyclidine, and dextrorphan inhibit NMDA receptors by this mechanism [35]. The only difference between the actions of Hyd-8 and phenytoin on AMPARs is that Hyd-8 forms a more stable complex with the receptor, manifested by slower recovery kinetics upon drug washout. For sodium-channel block, Hyd-8 shows all features typical of local anesthetics and anticonvulsants: weak tonic block of infrequent activations, frequency-dependent enhancement of block, and a negative shift of steady-state inactivation. The main difference between Hyd-8 and phenytoin is the stronger tonic block induced by Hyd-8. These results allow the conclusion that Hyd-8 retains all phenytoin features of sodium-channel and AMPAR blockade but has improved potency.

**Figure 9:**
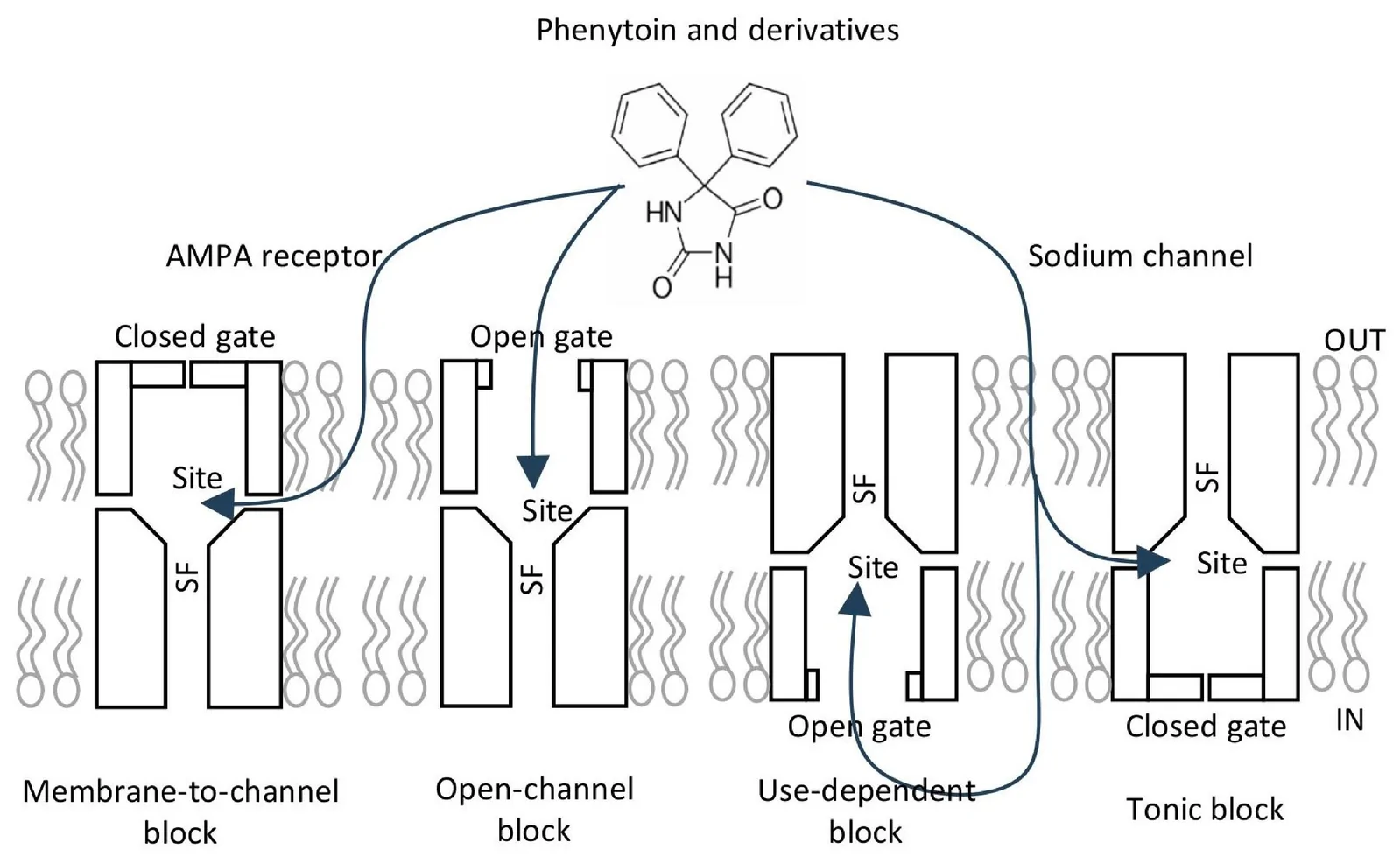
Schematic view of the mechanisms of block of AMPA receptors and sodium channels by phenytoin and its derivatives. The binding site is lοcated in the pore of both channels. In both cases, there is open-channel block through the open activation gate and closed-channel block through the membrane phase. The main difference is the opposite membrane topologies of the channels, which determines the locations of the selectivity filter (SF) and the activation gate.

General mechanism of action of phenytoin and its derivatives on two targets is illustrated in Figure 9. In both cases, the binding site is located in the central pore close to the selectivity filter. In both cases, there are distinct effects on the open and closed states. Closed-state block (called tonic block in the sodium-channel literature) occurs though the membrane phase. The drugs enter the channel and reach the binding site in the pore through the interface between subunits (repeats). The main difference is the opposite location of the activation gate, which is at the extracellular side in the AMPA receptor and at the intracellular side in the sodium channels. As a result, open-channel block by externally applied phenytoin occurs directly, whereas use-dependent block of sodium channels requires permeation through the membrane.

Taken together, our current findings indicate that pentobarbital and phenytoin may serve as structural prototypes for the development of novel compounds that simultaneously target voltage-gated sodium channels and AMPA receptors, which are among the key targets for potential anticonvulsive medications. This innovative approach may lead to the emergence of drugs that are effective against drug-resistant seizures. While our current work, which focuses on the effects of these two molecules on two types of ion channels, does not produce new anticonvulsants, it does suggest a promising avenue for future research. However, the clinical significance of these derivatives and their advantages over existing anti-seizure medications remain uncertain. Specifically, further studies are needed to investigate their pharmacokinetics, long-term safety, and in the vivo correlation between electrophysiological effects and anticonvulsant activity. The latter problem requires a comprehensive comparison of new phenytoin derivatives and conventional antiepileptics in various animal seizure models, and it is currently being conducted at our institution.

## 4. Materials and Methods

Phenytoin and its derivatives were synthesized for this work in the Department of Chemistry, Lomonosov Moscow State University, Russian Federation. NMR spectra were recorded on Bruker Avance 400 spectrometers (Bruker, Rheinstetten, Germany; 400.0 MHz for 1H; 100.6 MHz for ^13^C) at ambient temperature; the chemical shifts (δ scale) were referenced to the DMSO-d6 (1H: δ = 2.50 ppm, ^13^C: δ = 39.52 ppm). Chemical shifts (δ) are given in ppm; J values are given in Hz. A thin layer chromatographic method (TLC) was used on DC-Fertigfolien ALUGRAM pre-coated silica gel 60-F254 plates (MACHEREY-NAGEL GmbH & Co. KG, Düren, Germany); the detection was done with a UV lamp (254 and 365 nm) and chemical staining (5% aqueous solution of KMnO_4_). Column chromatography was performed on silica gel (230–400 mesh, Merck, Darmstadt, Germany). Details are presented in the Supplementary Material.

All experimental procedures were approved by the Animal Care and Use Committee of the I.M. Sechenov Institute of Evolutionary Physiology and Biochemistry of the Russian Academy of Sciences (protocol 2-3/2025, 27 February 2025). Outbred male Wistar rats (13–19 days old) were anesthetized with sevoflurane and then decapitated. Maximum efforts were made to minimize the number of animals used. Brains were removed quickly and cooled to 2–4 °C in an ice bath. Transverse hippocampal slices (250 µm thick) were prepared using a vibratome (Campden Instruments Ltd., Loughborough, UK) and stored in a solution containing (in mM): 124 NaCl, 5 KCl, 1.3 CaCl_2_, 2.0 MgSO_4_, 26 NaHCO_3_, 1.24 NaH_2_PO_4_, and 10 D-glucose, aerated with carbogen (95% O_2_, 5% CO_2_).

Single neurons were freed from slices by vibrodissociation [36,37]. The antagonism of CP-AMPARs was studied on striatal giant interneurons [38,39], which were identified by their shape and size. They have large (>25 µm) somata of polygonal shape, whereas principal cells are significantly smaller and nearly spherical. Previous work demonstrated that a non-desensitizing response to kainate in these neurons is mediated by GluA2-lacking AMPARs. The sensitivity to dicationic blockers like IEM-1460, IEM-1925 and polycationic toxins agrees with the data on recombinant receptors [8,40]. The currents demonstrate inward rectification and significant Ca^2+^ permeability [41,42]. The antagonism of CI-AMPARs and voltage-gated sodium channels (VGSCs) was studied on pyramidal neurons from the CA1 area of the hippocampus. These cells were isolated from the stratum pyramidale and distinguished from non-pyramidal cells on the basis of pyramidal-like somata and preserved apical dendrites. Kainate-induced currents in these neurons are virtually insensitive to cationic blockers [8,28]. The whole-cell patch-clamp technique was used for recording of membrane currents generated in response to applications of an agonist (AMPARs) or depolarization steps (VGSCs). Series resistance of about 20 MΩ was compensated by 70–80% and monitored during experiments. Currents were recorded using an EPC-8 amplifier (HEKA Electronics, Lambrecht, Germany), filtered at 5 kHz, sampled, and stored on a personal computer. Drugs were applied using the RSC-200 perfusion system (BioLogic Science Instruments, Claix, Seyssinet-Pariset, France) under computer control. The solution exchange time was ~200 ms. The extracellular solution contained (in mM): NaCl 143, KCl 5, MgSO_4_ 2, CaCl_2_ 2.5, D-glucose 18, HEPES 10 (pH was adjusted to 7.4 with HCl). For experiments with VGSCs, KCl was excluded, and CdCl_2_ (0.1 mM) was added. The pipette solution contained (in mM): CsF 100, CsCl 40, NaCl 5, CaCl_2_ 0.5, EGTA 5, and HEPES 10 (pH was adjusted to 7.2 with CsOH). Stock solutions of compounds were made in DMSO, and appropriate amounts of DMSO (maximum 0.6%) were added to control experimental solutions. Experiments were performed at room temperature (22–24 °C) at a holding voltage of −80 mV.

All experimental data are presented as mean ± SD estimated from at least four experiments. The significance of the effects was tested with *t*-tests or one-way ANOVA with Tukey’s post hoc test. Differences were considered significant at *p* < 0.05. Data were analyzed using Origin 2019b 9.65 software (OriginLab Corp., Northampton, MA, USA). Concentration dependencies were fitted with the Hill equation. In some cases, estimation of the entire concentration dependence in a single experiment was not possible. The data from different cells (*n* ≥ 4 for each concentration) were pooled and fitted. Approximation error values were taken as measures of precision. Inactivation curves for VGSCs were fitted with the Boltzmann equation:

(1) $$y\;=\;\frac{1}{1+exp(\frac{V-V_{1/2}}{k})},$$

where y is the normalized current, V the prepulse voltage, V_1/2_ the voltage of half-maximal inactivation, and k the slope factor. From the steady-state inactivation shift and the dose–response relationship, the apparent affinity for the inactivated state (K_i_) was calculated using the equation:

(2) $$K_{i}\;=\;\frac{C}{\frac{1+C/{IC}_{50}}{exp(\frac{\Delta V_{1/2}}{k})}-1},$$

where ΔV_1/2_ is the difference between the V_1/2_ values in presence and absence of compounds, k the slope of the steady-state inactivation curve, IC_50_ is the activity for the resting state taken from the dose response curve, and C represents the drug concentration responsible for the steady-state inactivation shift [27].

## Figures and Tables

**Table 1:** Inhibition of AMPA receptors by phenytoin and its derivatives; comparison with carbamazepine.

Compound	Inhibition by 100 μM, %	
	CI-AMPARs	CP-AMPARs
Carbamazepine	11 *	15 *
Phenytoin	78 ± 4 (*n* = 8)	35 ± 4 (*n* = 4)
Hyd-9	9 ± 5 (*n* = 11)	7 ± 3 (*n* = 4)
Hyd-1	10 ± 5 (*n* = 17)	10 ± 4 (*n* = 5)
Hyd-2	47 ± 3 (*n* = 12)	15 ± 3 (*n* = 4)
Hyd-5	15 ± 3 (*n* = 9)	5 ± 3 (*n* = 4)
Hyd-6	30 ± 7 (*n* = 8)	9 ± 5 (*n* = 4)
Hyd-8	85 ± 3 (*n* = 8)	58 ± 6 (*n* = 7)
Hyd-3	11 ± 4 (*n* = 8)	8 ± 4 (*n* = 5)
Hyd-4	16 ± 4 (*n* = 8)	9 ± 5 (*n* = 5)

* Data from [17].

**Table 2:** Inhibition of voltage-gated sodium channels by phenytoin and its derivatives; comparison with carbamazepine.

Compound	Inhibition by 100 μM, %	
	Tonic Block	Inactivation State
Carbamazepine	9 ± 2 (*n* = 4)	53 ± 5 (*n* = 4)
Phenytoin	20 ± 7 (*n* = 4)	65 ± 3 (*n* = 4)
Hyd-9	6 ± 1 (*n* = 4)	23 ± 3 (*n* = 4)
Hyd-1	8 ± 2 (*n* = 6)	26 ± 15 (*n* = 6)
Hyd-2	9 ± 5 (*n* = 7)	37 ± 10 (*n* = 6)
Hyd-5	7 ± 3 (*n* = 5)	32 ± 12 (*n* = 4)
Hyd-6	13 ± 7 (*n* = 6)	45 ± 14 (*n* = 6)
Hyd-8	28 ± 7 (*n* = 6)	68 ± 7 (*n* = 5)
Hyd-3	4 ± 1 (*n* = 6)	15 ± 7 (*n* = 6)
Hyd-4	6 ± 4 (*n* = 6)	24 ± 6 (*n* = 6)

## Data Availability

Original data are available on request.

## Supplementary Materials

The following supporting information can be downloaded at https://www.mdpi.com/article/10.3390/ijms27177723/s1. References [43,44,45,46,47,48,49,50,51,52] are cited in the supplementary materials.

## Abbreviations

The following abbreviations are used in this manuscript:

AMPA	α-Amino-3-hydroxy-5-methyl-4-isoxazolepropionic acid
CI-AMPARs	Calcium-impermeable AMPA receptors
CP-AMPARs	Calcium-permeable AMPA receptors
DNQX	6,7-Dinitroquinoxaline-2,3-dione
EGTA	Ethylene-glycol-bis(β-aminoethyl-ether)-N,N,N′,N′-tetraacetic acid
NMDARs	N-Methyl-D-aspartate receptors
VGSCs	Voltage-gated sodium channels
