# Supplementary material for: Phenytoin Derivatives as Antagonists of AMPA Receptors and Voltage-Gated Sodium Channels: A Structure–Function Study
Source: Int J Mol Sci. 2026 Aug 28;27(17):7723. doi: 10.3390/ijms27177723 (PMC13566764; doi:10.3390/ijms27177723)
Supplement: Supplementary file 1 [file ijms-27-07723-s001.zip › ijms-4479956-supplementary.pdf]

SUPPLEMENTARY MATERIAL

Phenytoin derivatives as antagonists of AMPA receptors and voltage-gated sodium channels: structure-function study

Arseniy S. Zhigulin [1], Maxim V. Nikolaev [1], Mikhail Y. Dron [1], Dmitry A. Vasilenko [2], Oleg I. Barygin [1] and Denis B. Tikhonov [1,*]

1 Sechenov Institute of Evolutionary Physiology and Biochemistry, Russian Academy of Sciences, 194223 Saint Petersburg, Russia; arseniy.zhigulin@yandex.ru (A.S.Z.); fmedfstud@gmail.com (M.V.N.); neuro.mike@yahoo.com (M.Y.D.); oleg_barygin@mail.ru (O.I.B.)

2 Department of Chemistry, Lomonosov Moscow State University, 119991 Moscow, Russia; vda-ga@yandex.ru (D.A.V.)

* Correspondence: denistikhonov2002@yahoo.com

Synthesis of phenytoin and its derivatives

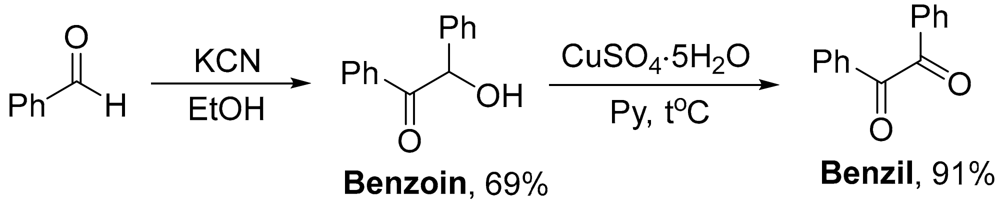

Scheme S1. Synthesis of benzyl

Benzoin was prepared according to the literature procedure [43].

A 250-mL round-bottom flask was charged with 115 mL of 96% ethanol and 80 mL (83.2 g, 0.785 mol) of benzaldehyde (purified by vacuum distillation). A solution of 12 g (0.185 mol) of KCN in 80 mL of distilled water was added, and the mixture was stirred for 3 h under reflux. The resulting brownish solution was left to stand in the fridge for 14 hours. The precipitated crude product was filtered off and then crystallized using 450 ml of 96% ethanol. Yield 57.7 g (69%), white crystals.

Benzil was prepared according to the literature procedure [44].

A solution of 139 g (0.56 mol) of $CuSO_4 \cdot 5H_2O$ in the mixture of 131 mL of pyridine and 108 mL of water was prepared by heating on a steam bath. Benzoin (57.7 g, 0.27 mol) was added to the resulting solution, and the mixture was heated on the steam bath for 2 hours. The reaction mixture turned dark green, and the product was collected in the upper layer as oil, which solidifies upon cooling. The solidified benzil was filtered, washed with water, and dried. The product was recrystallized from carbon tetrachloride. Yield 52.0 g (91 %), yellow-green crystals.

Phenytoin was obtained using the modified literature procedure [45].

A solution of benzil (10.6 g, 0.05 mol) in 150 mL of 96% ethanol was placed in a 500 ml round-bottomed flask equipped with a reflux condenser. Urea (6.0 g, 0.1 mol) and 30 mL of 30% aqueous NaOH were sequentially added to the solution, and the resulting mixture was heated for 3 h under reflux with stirring. Afterwards, the mixture was cooled to room temperature and poured into 250 mL of distilled cold water. Vigorously stirred for 15 min resulted in the formation of a solid, which was filtered off. Then concentrated hydrochloric acid was added to the filtrate (on cooling with ice) to obtain a strongly acidic reaction. The colorless precipitate was filtered off, which was recrystallized from 96% ethanol. Yield 4.8 g (38 %), colorless needles.

^{1}H NMR (DMSO-d6, 400 MHz): δ 11.18 (1H, s, NH), 9.38 (1H, s, NH), 7.44-7.36 (8H, m, Ph), 7.36-7.29 (2H, m, Ph).

^{13}C{^{1}H} NMR (DMSO-d6, 100.6 MHz): δ 175.0, 156.2, 140.0, 128.6, 128.1, 126.7, 70.4.

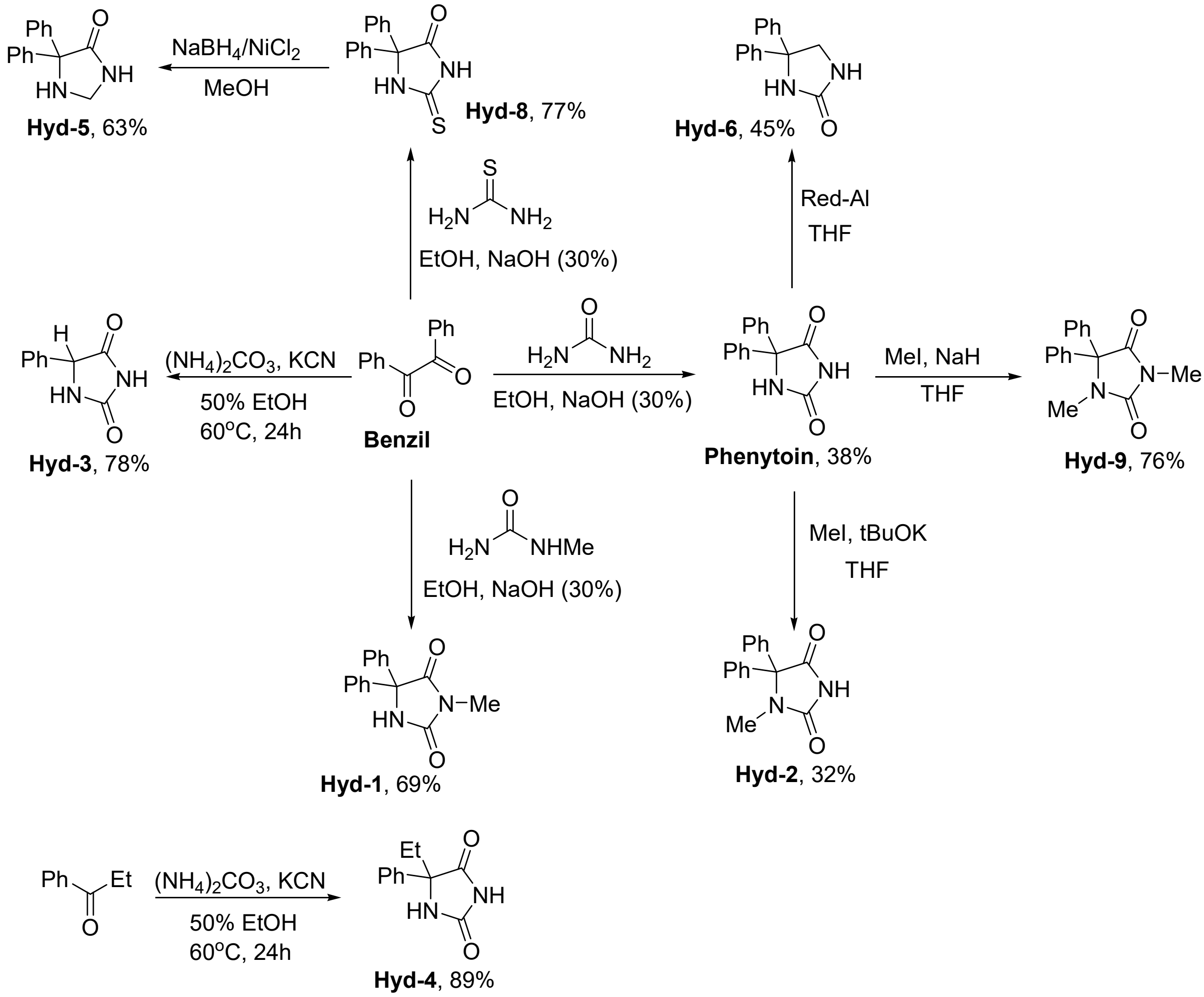

Scheme S2. Synthesis of phenytoin and its derivatives

3-Methyl-5,5-diphenylimidazolidine-2,4-diones (Hyd-1) was obtained using the modified literature procedure [45].

A solution of benzil (6.60 g, 0.031 mol) in 100 mL of 96% ethanol was placed in a 500 mL round-bottomed flask equipped with a reflux condenser. 1-Methylurea (4.65 g, 0.063 mol) and 19 mL of

30% aqueous NaOH were sequentially added to the solution, and the resulting mixture was heated for 3 h under reflux with stirring. Afterwards, the mixture was cooled to room temperature and poured into 160 ml of distilled cold water. Vigorously stirred for 15 min resulted in the formation of a solid, which was filtered off. Then concentrated hydrochloric acid was added to the filtrate (on cooling with ice) to obtain a strongly acidic reaction. The colorless precipitate was filtered off and repeatedly (2 times) recrystallized from 96% ethanol. Yield 5.8 g (69 %), pale yellow solid.

^{1}H NMR (DMSO-d6, 400 MHz): δ 9.65 (1H, s, NH), 7.43-7.30 (10H, m, Ph), 2.93 (3H, s, CH_3).

^{13}C{^{1}H} NMR (DMSO-d6, 100.6 MHz): δ 173.3, 155.6, 139.7, 128.6, 128.2, 126.7, 69.2, 24.6.

1-Methyl-5,5-diphenylimidazolidine-2,4-dione (Hyd-2) was prepared according to the literature procedure [46]

To a solution of phenytoin (500 mg, 1.98 mmol) in THF (10 mL), a solution of tBuOK (318 mg, 3.97 mmol) in THF (4.0 mL) was added at r.t. After 3 min CH_3I (150 μL, 2.38 mmol) in THF (0.5 mL) was added at r.t. and the mixture was stirred at r.t. for 1 h. One molar HCl (10 mL) was added and the whole was extracted with EtOAc (4 × 50). The combined organic layers were washed with brine (1 × 10 mL) and dried over $MgSO_4$. The solvent was evaporated in vacuo, and the residue was separated by column chromatography (petroleum ether/EtOAc = 5/2). Three different fractions were isolated containing **Hyd-9** (150 mg, 27%, colorless solid, R_f = 0.52), **Hyd-1** (95 mg, 18%, pale yellow solid, R_f = 0.36) and **Hyd-2** (169 mg, 32%, light yellow solid, R_f = 0.25) subsequently.

^{1}H NMR (DMSO-d6, 400 MHz): δ 11.40 (1H, s, NH), 7.50-7.39 (6H, m, Ph), 7.23-7.17 (4H, m, Ph), 2.62 (3H, s, CH_3).

^{13}C{^{1}H} NMR (DMSO-d6, 100.6 MHz): δ 174.4, 155.7, 136.6, 128.8, 128.7, 127.9, 74.7, 26.1.

5-Phenylimidazolidine-2,4-dione (Hyd-3) was prepared according to the literature procedure [47]

KCN (2.6 g, 0.04 mol) and $(NH_4)_2CO_3$ (7.7 g, 0.08 mol) were sequentially added to a solution of benzil (4.2 g, 0.02 mol) in 50 mL of 50% ethanol. The resulting mixture was stirred at 60 ºC for 24 h, after which the solution was filtered. The pH of the aqueous filtrate solution was adjusted to pH 2-3 by acidification with hydrochloric acid. The product was filtered off and recrystallized from ethanol (96%). Yield 2.7 g (78 %), colorless solid.

^{1}H NMR (DMSO-d6, 400 MHz): δ 10.79 (1H, s, NH), 8.41 (1H, s, NH), 7.44-7.38 (2H, m, Ph), 7.38-7.29 (3H, m, Ph), 5.16 (1H, s, CH).

^{13}C{^{1}H} NMR (DMSO-d6, 100.6 MHz): δ 174.2, 157.5, 136.1, 128.7, 128.3, 126.7, 61.2.

5-Ethyl-5-phenylimidazolidine-2,4-dione (Hyd-4) was prepared according to the literature procedure [48]

KCN (2.6 g, 0.04 mol) and $(NH_4)_2CO_3$ (7.7 g, 0.08 mol) were sequentially added to a solution of propiophenone (2.66 mL, 2.68 g, 0.02 mol) in 50 mL of 50% ethanol. The resulting mixture was stirred at 60 ºC for 24 h, after which the solution was concentrated to approximately two-third of the initial volume. Then it was cooled to 0ºC (ice-base) and filtered. The obtained product was

dissolved in 5% of NaOH solution, filtered, reprecipitated by acidification with hydrochloric acid and recrystallized from ethanol (60%). Yield 3.6 g (89 %), colorless solid.

^{1}H NMR (DMSO-d6, 400 MHz): δ 10.77 (1H, s, NH), 8.65 (1H, s, NH), 7.53-7.47 (2H, m, Ph), 7.43-7.35 (2H, m, Ph), 7.35-7.29 (1H, m, Ph), 2.07 (1H, dq, *J* = 7.3 Hz, *J* = 14.0 Hz, CH_2), 1.89 (1H, dq, *J* = 7.3 Hz, *J* = 14.0 Hz, CH_2), 0.81 (3H, t, *J* = 7.3 Hz, CH_3).

^{13}C{^{1}H} NMR (DMSO-d6, 100.6 MHz): δ 176.3, 156.7, 139.1, 128.5, 127.8, 125.4, 68.0, 31.2, 8.1.

5,5-Diphenyl-2-thioxoimidazolidin-4-one (Hyd-8) was obtained using the modified literature procedure [49].

Thiourea (8.0 g, 0.1 mol) and 30 mL of 30% aqueous NaOH were sequentially added to a solution of benzil (10.3 g, 0.05 mol) in 150 mL of 96% ethanol. The resulting mixture was refluxed for 2 h. Then it was allowed to cool and poured into crushed ice with constant stirring. The obtained insoluble by-product was removed by filtration. The filtrate was acidified with conc. HCl and the obtained precipitated solid was filtered, dried and recrystallized from ethanol (96%). Yield 10.3 g (77 %), colorless needles.

^{1}H NMR (DMSO-d6, 400 MHz): δ 12.18 (1H, s, NH), 11.35 (1H, s, NH), 7.48-7.35 (6H, m, Ph), 7.35-7.27 (4H, m, Ph).

^{13}C{^{1}H} NMR (DMSO-d6, 100.6 MHz): δ 181.4, 175.3, 138.4, 128.8, 128.5, 126.6, 73.0.

5,5-Diphenyl-4-imidazolidone (Hyd-5) was prepared according to the literature procedure [50].

A solution of **Hyd-8** (4.0 g, 0.015 mol) in 200 mL of dry methanol and anhydrous nickel chloride (15.6 g, 0.119 mol) were placed in a 500 ml round-bottomed flask equipped with a reflux condenser and a $CaCl_2$ guard tube. Sodium borohydride (4.6 g, 0.119 mol) was added very cautiously while stirring the solution vigorously. The reaction progress was monitored by TLC using petroleum ether-ethyl acetate (2:3) as eluent. After the disappearance of the starting material, the reaction mixture was filtered through a layer of silica gel (40 g), which was additionally washed with methanol (300 mL). The combined filtrate was diluted with water (500 mL) and extracted with ethyl acetate (5×100 mL). The combined organic layers were dried over anhydrous $MgSO_4$ and evaporated under vacuum. The resulting residue was recrystallized two times from ethanol (96%) and then from EtOAc. Yield 2.2 g (63%), colorless needle.

^{1}H NMR (DMSO-d6, 400 MHz): δ 8.38 (1H, s, NH), 7.52-7.45 (4H, m, Ph), 7.36-7.28 (4H, m, Ph), 7.28-7.22 (2H, m, Ph), 4.17 (2H, d, J = 8.0 Hz, CH_2), 3.92 (1H, br. t, J = 8.0 Hz, NH).

^{13}C{^{1}H} NMR (DMSO-d6, 100.6 MHz): δ 176.6, 142.2, 128.0, 127.2, 127.1, 68.3, 56.5.

4,4-Diphenylimidazolidin-2-one (Hyd-6) was obtained using the modified literature procedure [51].

A solution of phenytoin (0.50 g, 1.98 mmol) in 13 mL of dry THF was added dropwise to a solution of Red-Al (70% sol. in toluene (3.5 M), 1.98 mL, 6.93 mmol) in 6 mL of dry THF. The mixture was refluxed for 5 h. Excess of hydride was destroyed by the addition of a saturated solution of Na_2SO_4. The resulting precipitation of Al_2O_3 was decanted and washed with THF (2 × 10 mL).

The combined organic layers were washed with brine (1 × 20 mL), dried over Na_2SO_4 and evaporated under vacuum. The resulting residue after ether trituration was recrystallized two times from EtOAc. Yield 210 mg (45%), colorless needle.

^{1}H NMR (DMSO-d6, 400 MHz): δ 7.89 (1H, s, NH), 7.41-7.35 (4H, m, Ph), 7.35-7.28 (4H, m, Ph), 7.25-7.19 (2H, m, Ph), 6.45 (1H, br. s, NH), 3.89 (2H, s, CH_2).

^{13}C{^{1}H} NMR (DMSO-d6, 100.6 MHz): δ 162.3, 146.0, 128.2, 126.8, 125.8, 65.8, 53.3.

1,3-Dimethyl-5,5-diphenylimidazolidine-2,4-dione (Hyd-9) was obtained by using the modified procedure [52].

A solution of phenytoin (1.00 g, 3.97 mmol) in THF (10 mL) was added to a suspension of NaH (60%, 380 mg, 9.50 mmol) in THF (4 mL) at 0◦C within 5 min and the resulted mixture was stirred at room temperature for 1 h. After cooling to 0ºC, CH_3I (1.50 mL, 24.2 mmol) was added and the whole was stirred at room temperature for 6 h. Then the reaction mixture was diluted with the excess of 10% HCl (10 mL) and extracted with EtOAc (4 × 50 mL). The combined organic layer was washed with brine (1 × 5 mL) and dried over $MgSO_4$. The solvent was evaporated in vacuo, and the crude product was recrystallized from EtOAc. Yield 0.84 g (76%), colorless solid.

^{1}H NMR (DMSO-d6, 400 MHz): δ 7.49-7.40 (6H, m, Ph), 7.24-7.17 (4H, m, Ph), 3.00 (3H, s, CH_3), 2.67 (3H, s, CH_3).

^{13}C{^{1}H} NMR (DMSO-d6, 100.6 MHz): δ 173.1, 155.5, 136.3, 128.8, 128.0, 73.9, 26.5, 25.1.

References

43. Alia K.; Reghioua A.; Atia D.; Mbuvi H.M.; Moussaoui Y.; Jawad A.H. Magnetic chitosan-benzoin Schiff's base/fly ash for reactive blue 19 dye removal: Characterization and parametric optimization, *Inorg. Chem. Commun.*, **2024**, *169*, 112988. https://doi.org/10.1016/j.inoche.2024.112988.

44. Clarke H.T.; Dreger E.E. Benzil, *Org. Synth.*, **1926**, *6*, 6-7. https://doi.org/10.15227/orgsyn.006.0006.

45. Fetzer C.; Korotkov V.S.; Sieber S.A. Hydantoin analogs inhibit the fully assembled ClpXP protease without affecting the individual peptidase and chaperone domains, *Org. Biomol. Chem.*, **2019**, *17*, 7124-7127. https://doi.org/10.1039/c9ob01339c.

46. Shintani Y.; Kato K.; Kawami M.; Takano M.; Kumamoto T., D Direct N^1-Selective Alkylation of Hydantoins Using Potassium Bases, *Chem. Pharm. Bull.*, **2021**, *69*, 407-410. https://doi.org/10.1248/cpb.c20-00857.

47 Mahmoodi N.O.; Khodaee Z. Evaluating the one-pot synthesis of hydantoins. *Arkivoc*, **2007**, 2007(iii), 29-36. https://doi.org/10.3998/ark.5550190.0008.304.

48 Trišović N.; Timić T.; Divljaković J.; Rogan J.; Poleti D.; Savić M.M.; Uščumlić G., Synthesis, structural and biological characterization of 5-phenylhydantoin derivatives as potential anticonvulsant agents, *Monatsh. Chem.*, **2012**, *143*, 1451-1457. https://doi.org/10.1007/s00706-012-0791-8.

49. Dangi R.R.; Hussain N.; Talesara G.L., Synthesis characterization and biological evaluation of some alkoxyphthalimide derivatives of 3-(4-substituted phenyl)-6,6-diphenyl-3,3a-dihydro-2H-imidazo[2,1-b]pyrazolo[3,4-d][1,3]thiazol-7(6H)-one, *Med. Chem. Res.*, **2010**, *20*, 1490-1498. https://doi.org/10.1007/s00044-010-9392-4.

50. Khurana J.M.; Agrawal A.; Bansal G. 5,5-diaryl and 5-alkyl-3-phenyl-4-imidazolidones: A novel synthesis, *J. Heterocycl. Chem.*, **2009**, *46*, 1007-1010. https://doi.org/10.1002/jhet.165.

51 Kieseritzky F.; Lindström J., Aziridines in one step from hydantoins via Red-Al mediated ring-contraction, *Tetrahedron Lett.*, **2011**, *52*, 4558-4561. https://doi.org/10.1016/j.tetlet.2011.06.092.

52. Moribe K.; Ogino A.; Kumamoto T.; Ishikawa T.; Limwikrant W.; Higashi K.; Yamamoto K., Mechanism of Nanoparticle Formation from Ternary Coground Phenytoin and Its Derivatives, *J. Pharm. Sci.*, **2012**, *101*, 3413-3424. https://doi.org/10.1002/jps.23141.

Spectral copies of ^{1}H and ^{13}C NMR of compounds

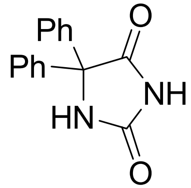

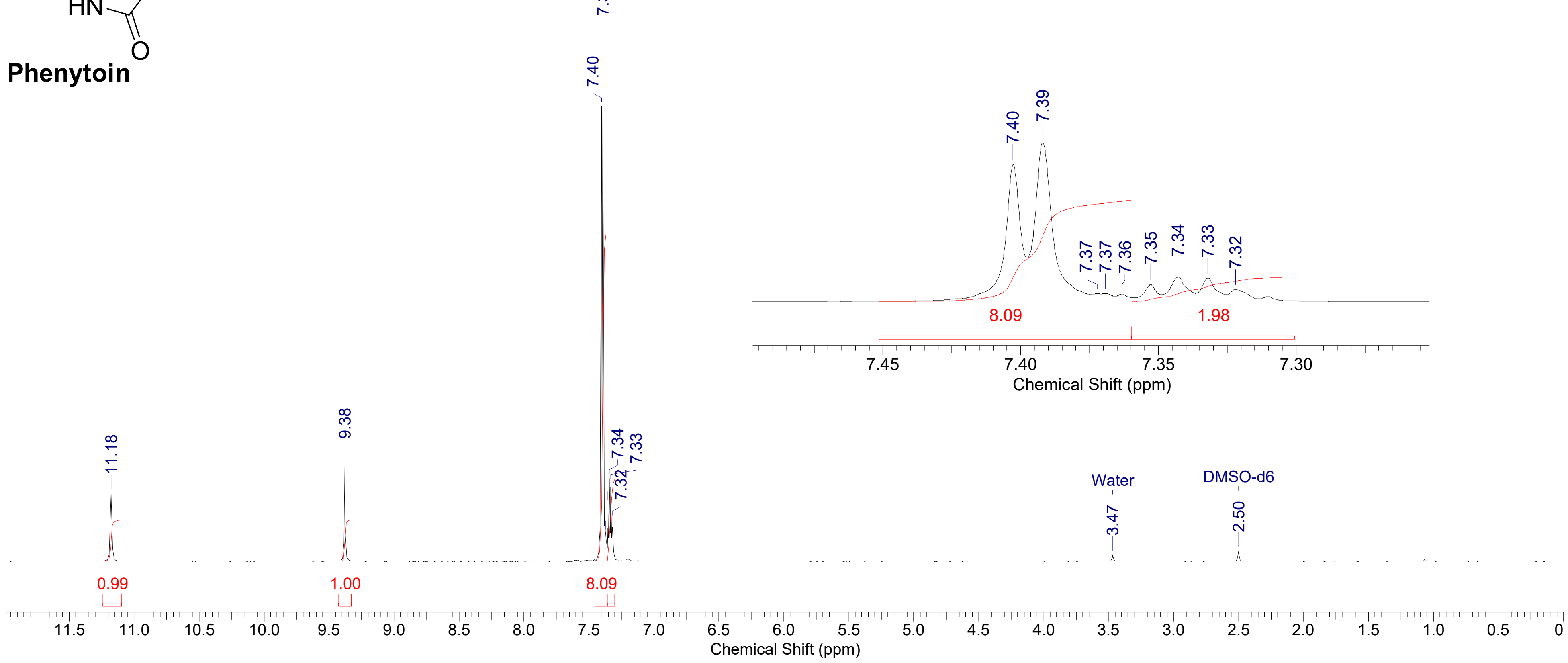

Figure S1. ^{1}H NMR (400 MHz, DMSO-d6) spectrum for phenytoin

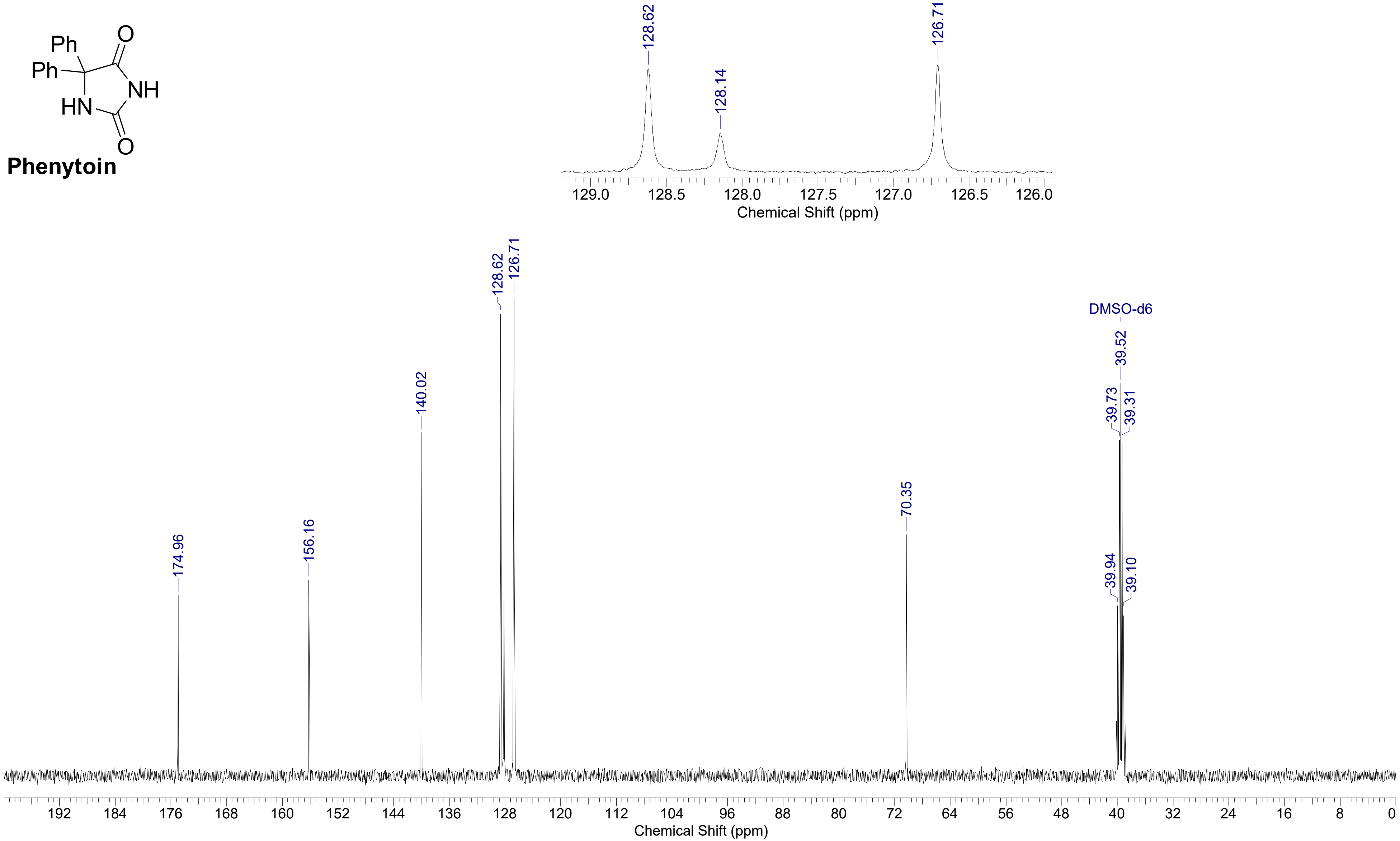

Figure S2. ^{13}C NMR (100.6 MHz, DMSO-d6) spectrum for phenytoin

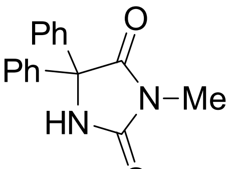

Hyd-1

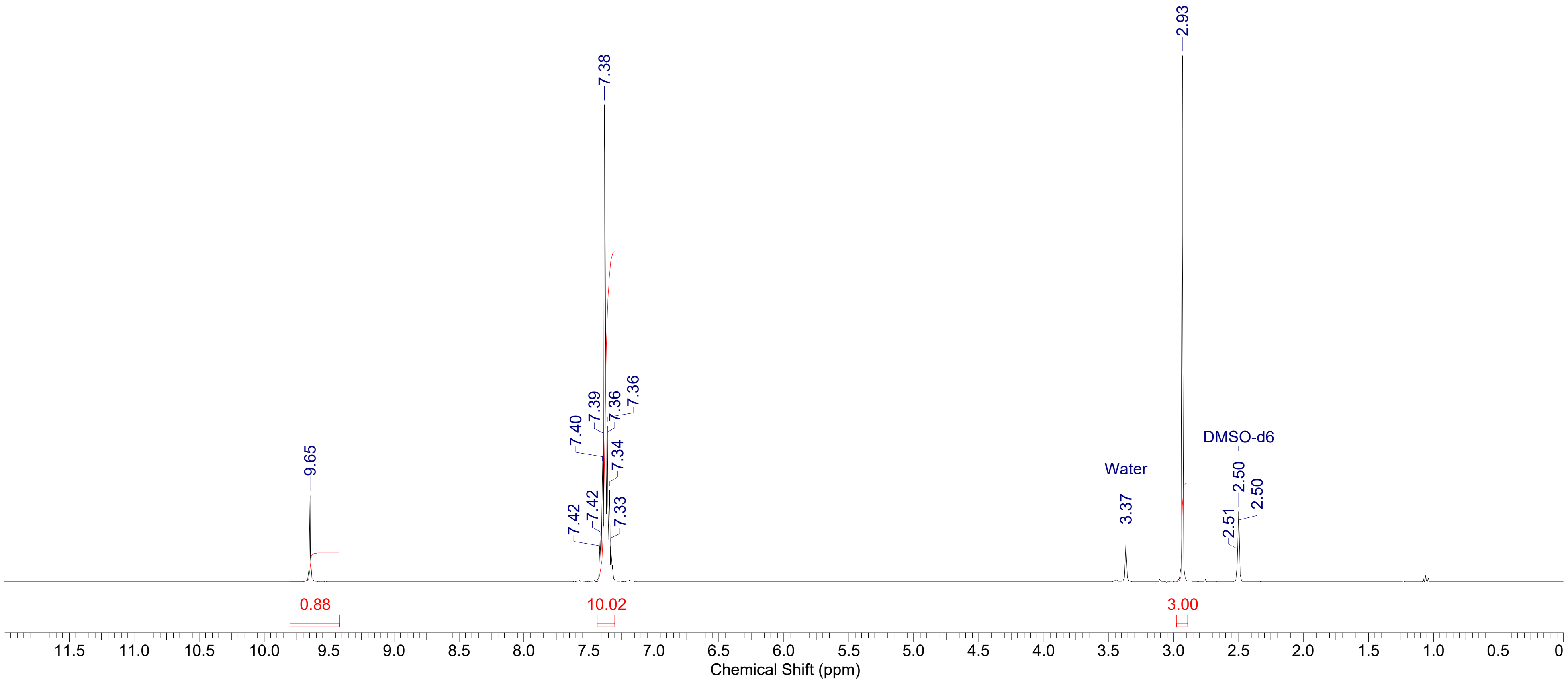

Figure S3. ^{1}H NMR (400MHz, DMSO-d6) spectrum for **Hyd-1**

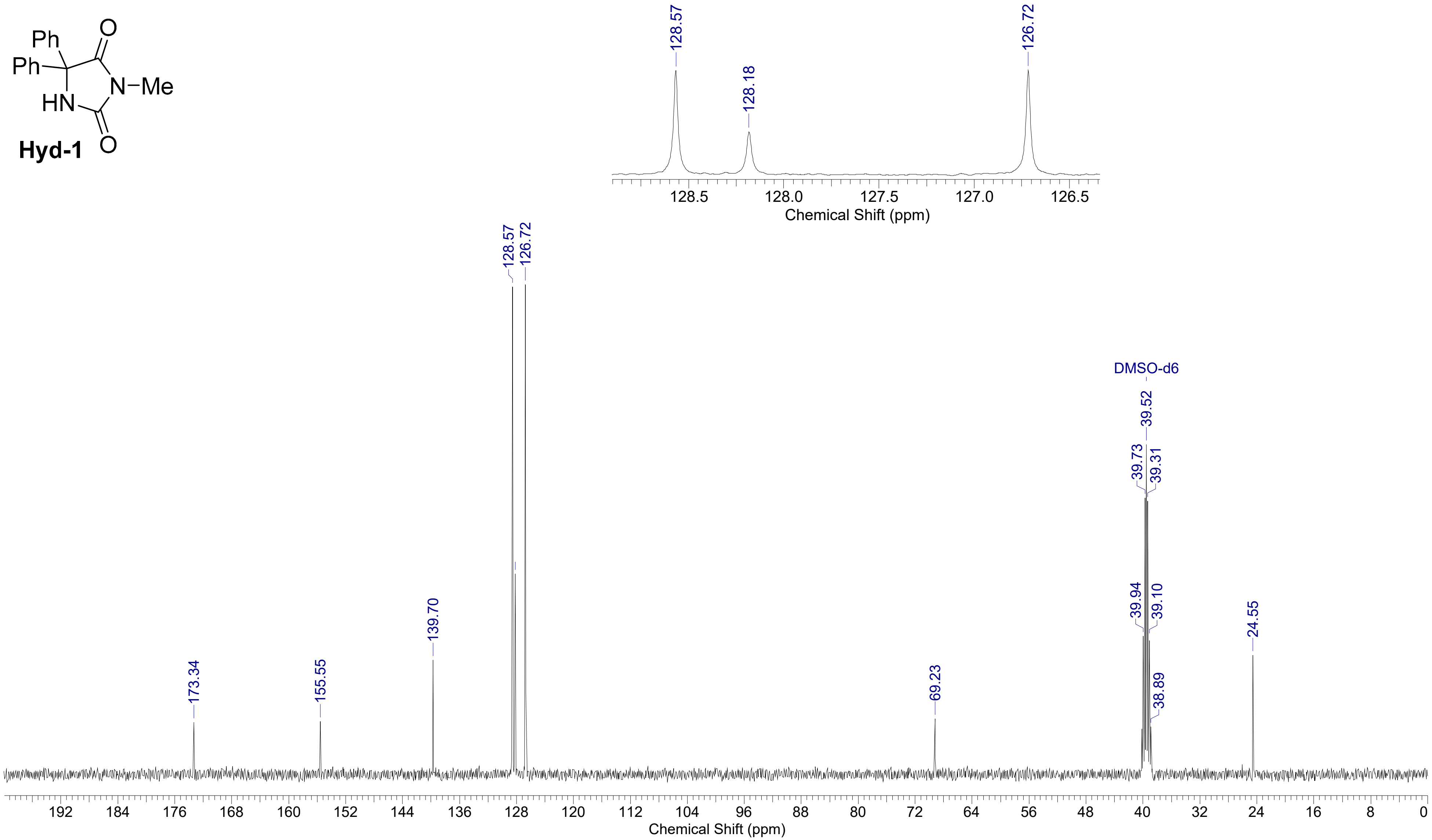

Figure S4. ^{13}C NMR (100.6 MHz, DMSO-d6) spectrum for **Hyd-1**

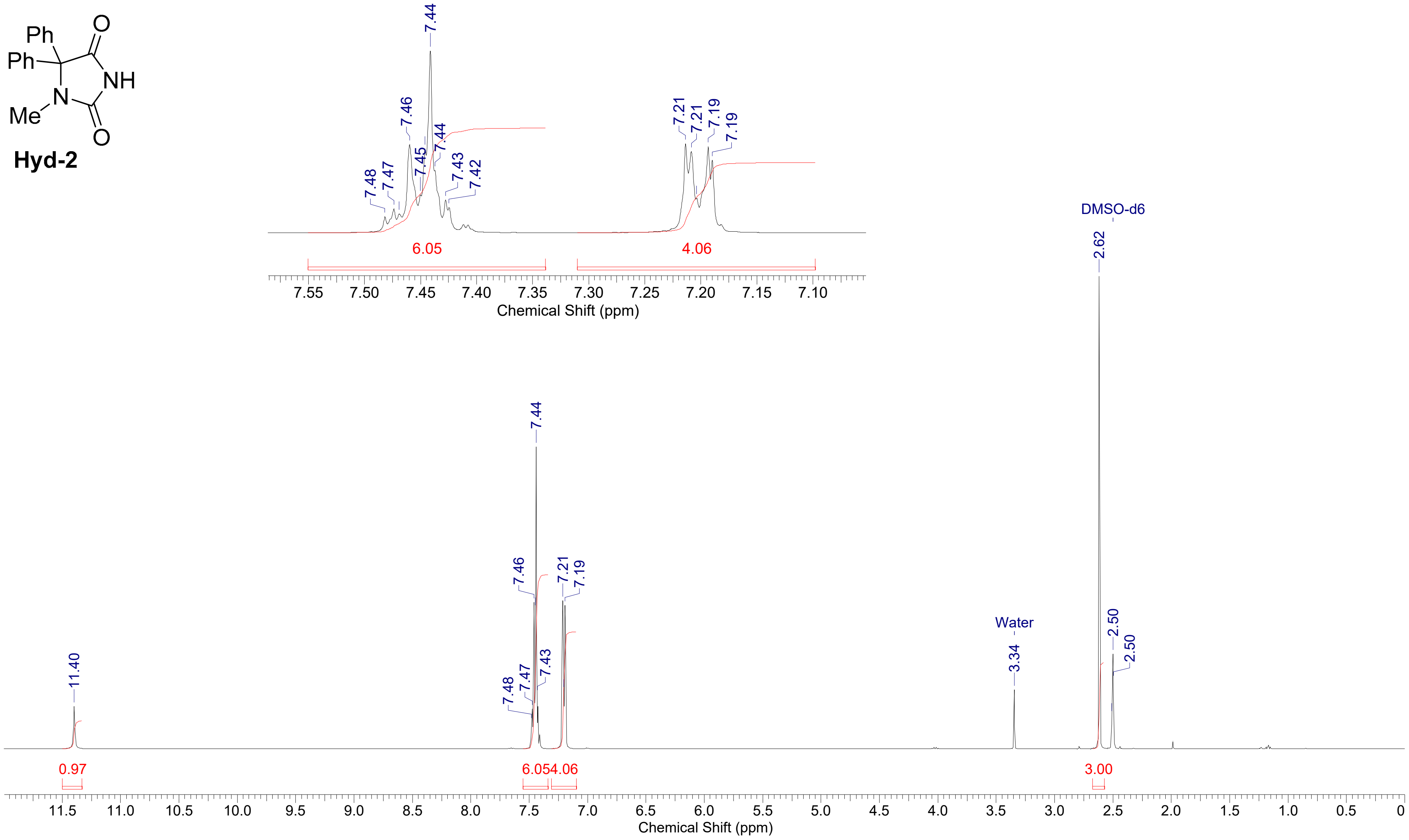

Figure S5. ^{1}H NMR (400 MHz, DMSO-d6) spectrum for **Hyd-2**

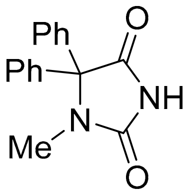

Hyd-2

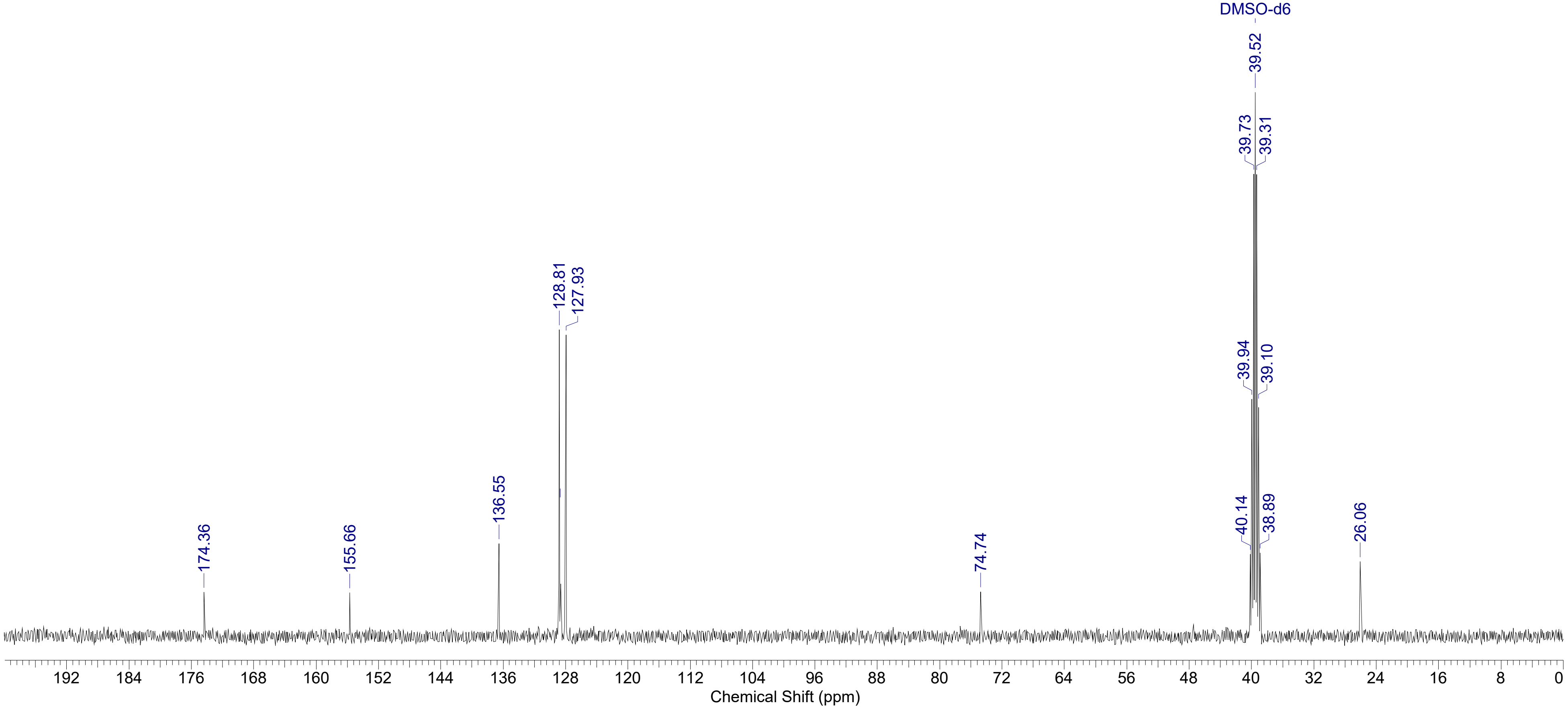

Figure S6. ^{13}C NMR (100.6 MHz, DMSO-d6) spectrum for **Hyd-2**

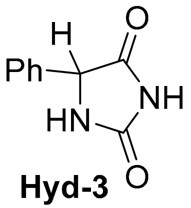

Hyd-3

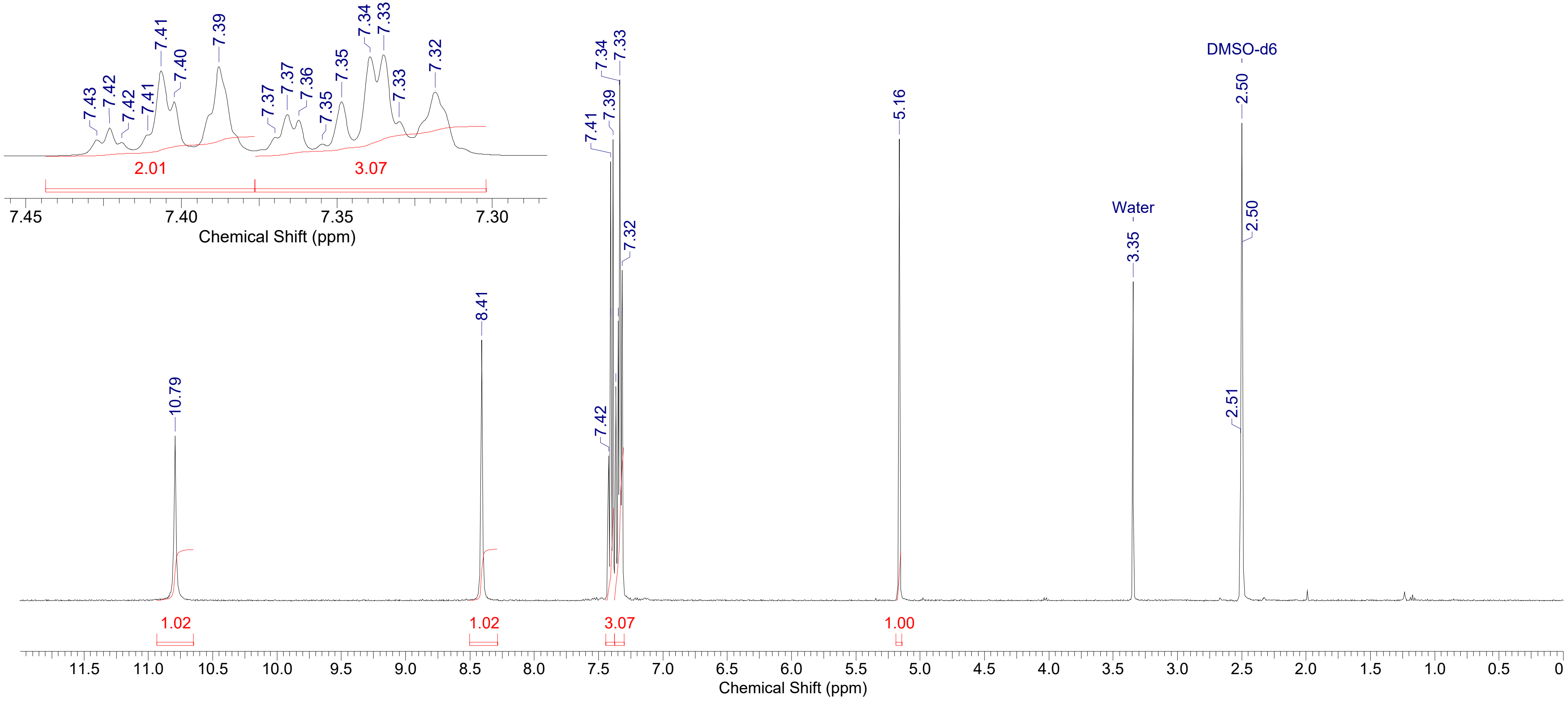

Figure S7. ^{1}H NMR (400 MHz, DMSO-d6) spectrum for **Hyd-3**

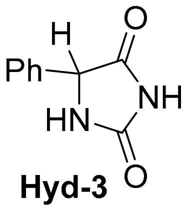

Hyd-3

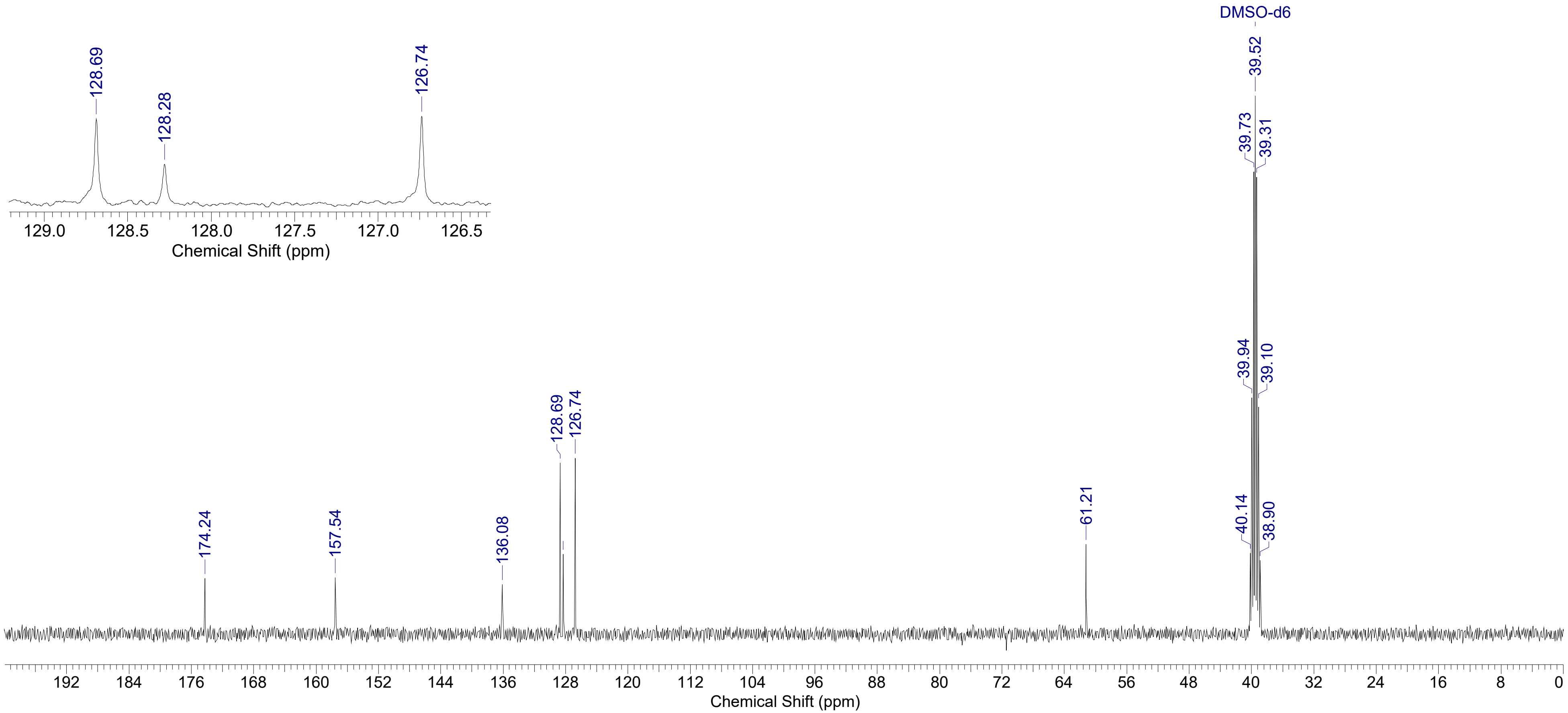

Figure S8. ^{13}C NMR (100.6 MHz, DMSO-d6) spectrum for **Hyd-3**

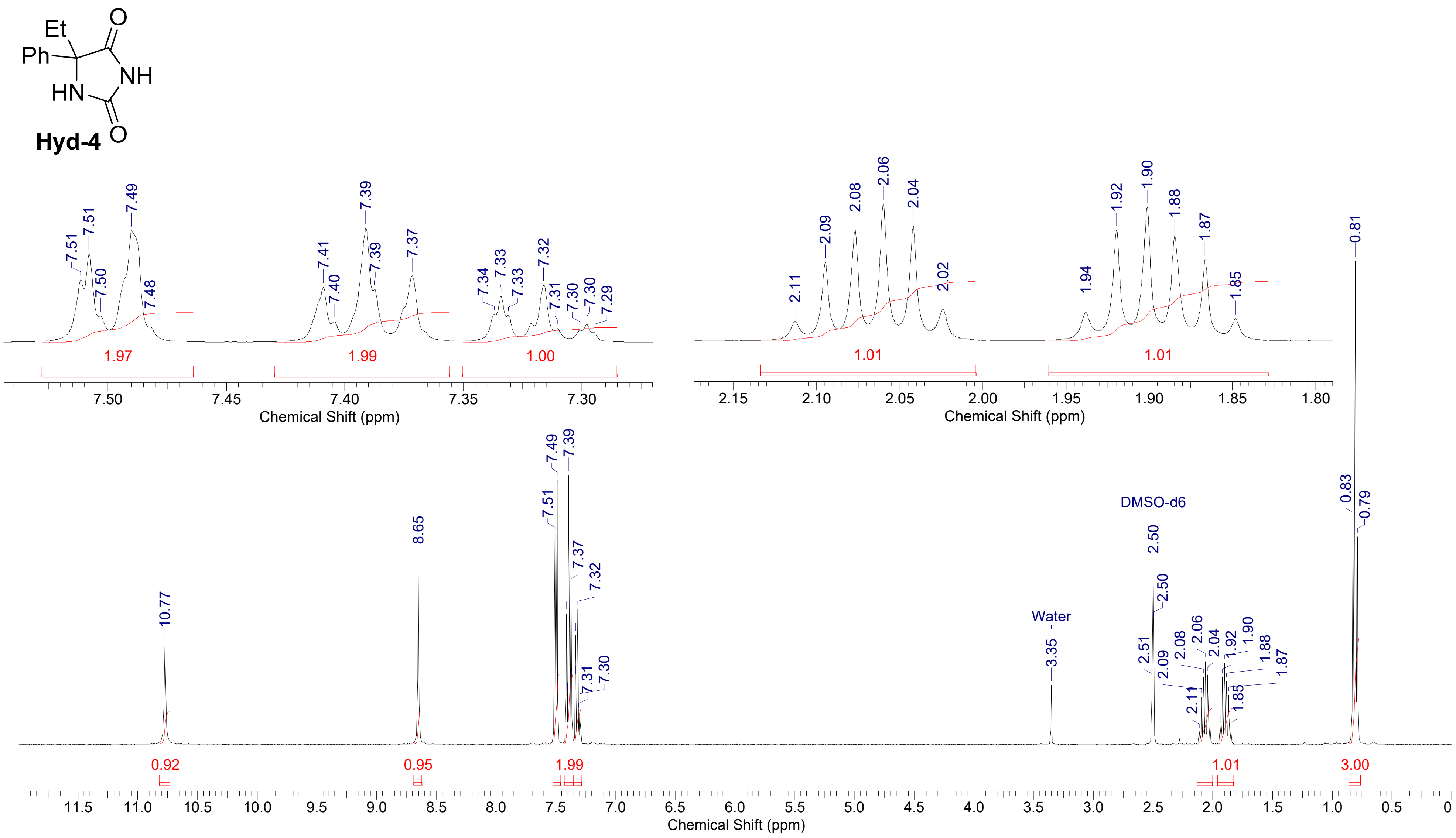

Figure S9. ^{1}H NMR (400 MHz, DMSO-d6) spectrum for **Hyd-4**

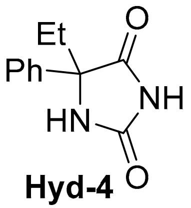

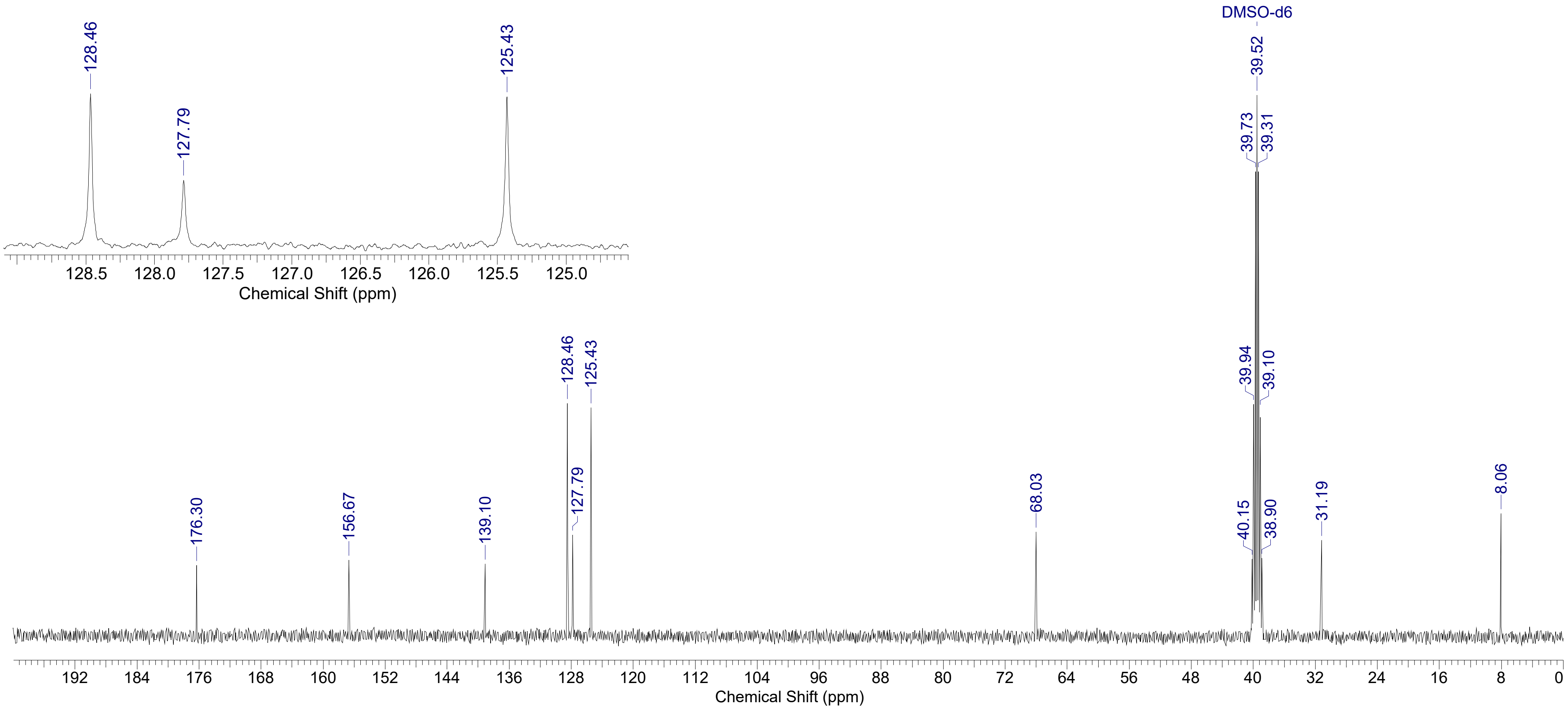

Figure S10. ^{13}C NMR (100.6 MHz, DMSO-d6) spectrum for **Hyd-4**

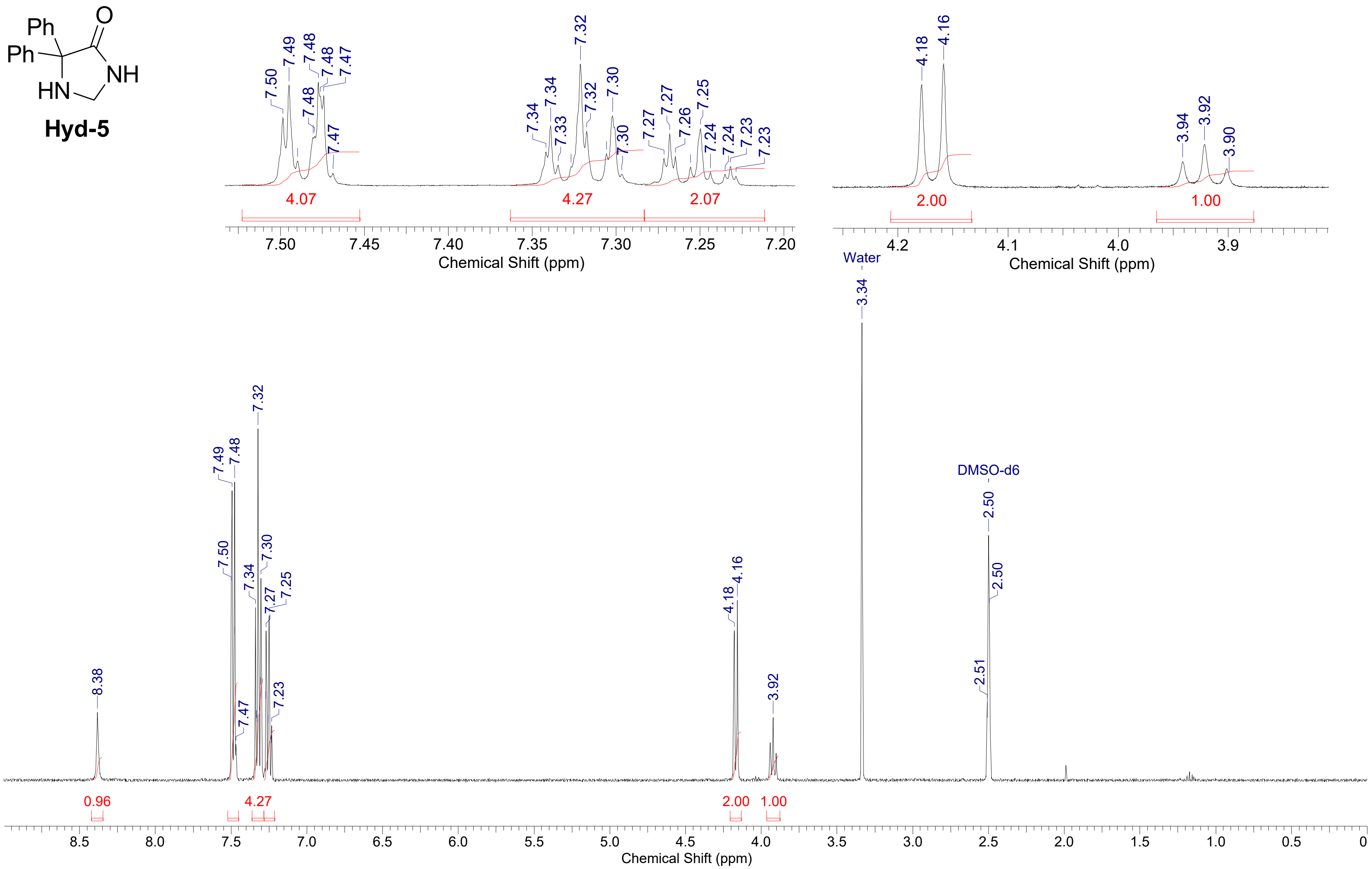

Figure S11. ^{1}H NMR (400 MHz, DMSO-d6) spectrum for **Hyd-5**

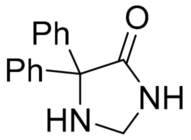

Hyd-5

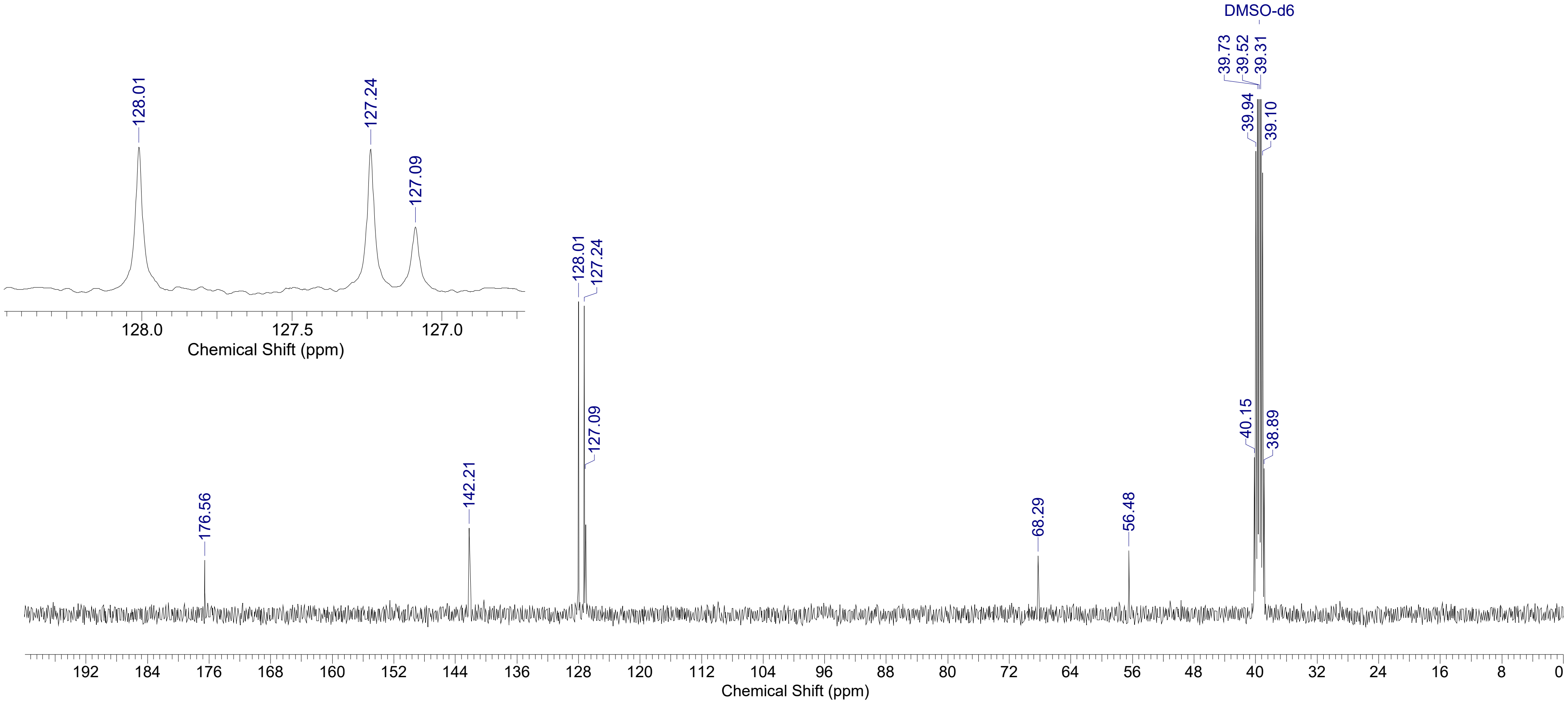

Figure S12. ^{13}C NMR (100.6 MHz, DMSO-d6) spectrum for **Hyd-5**

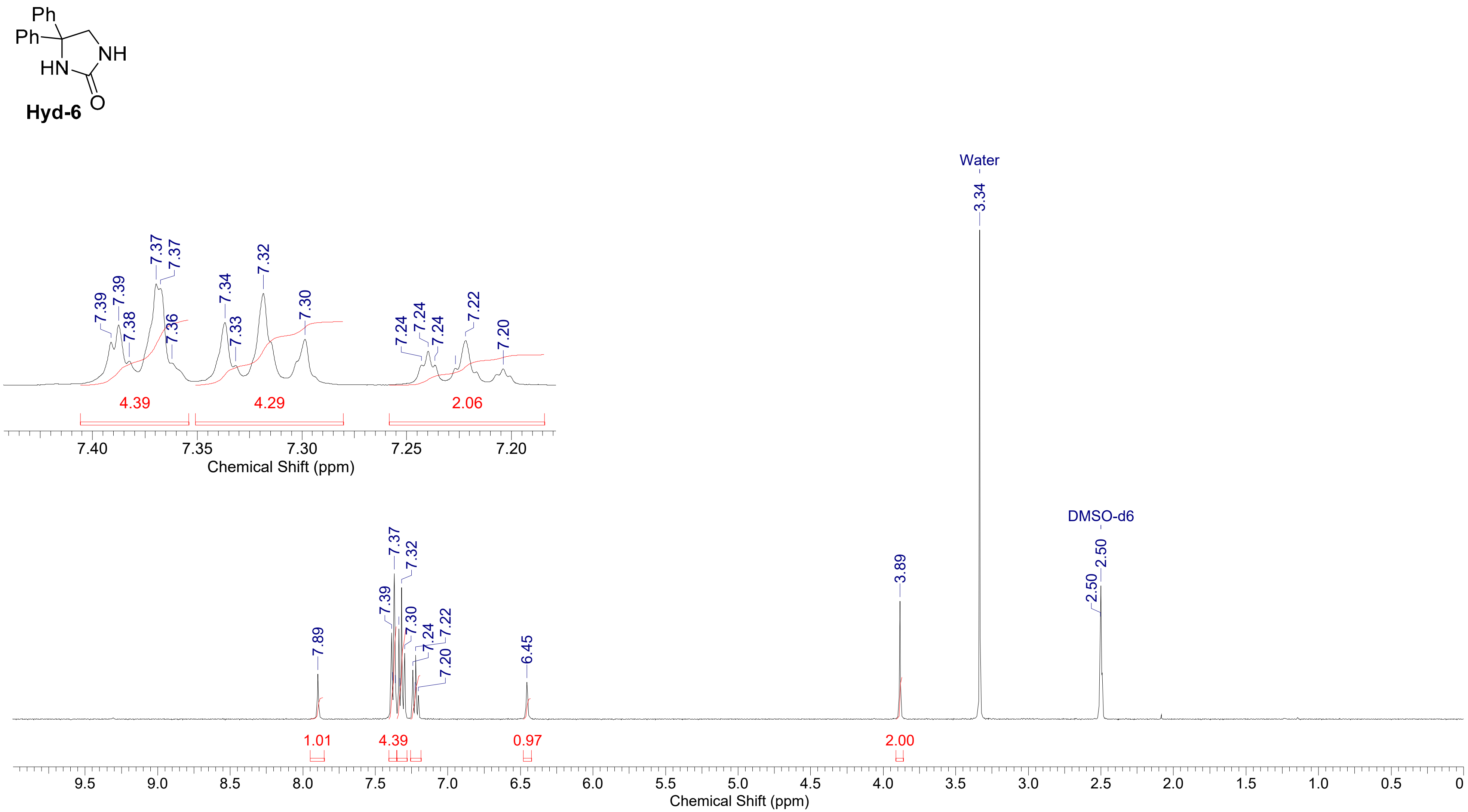

Figure S13. ^{1}H NMR (400 MHz, DMSO-d6) spectrum for **Hyd-6**

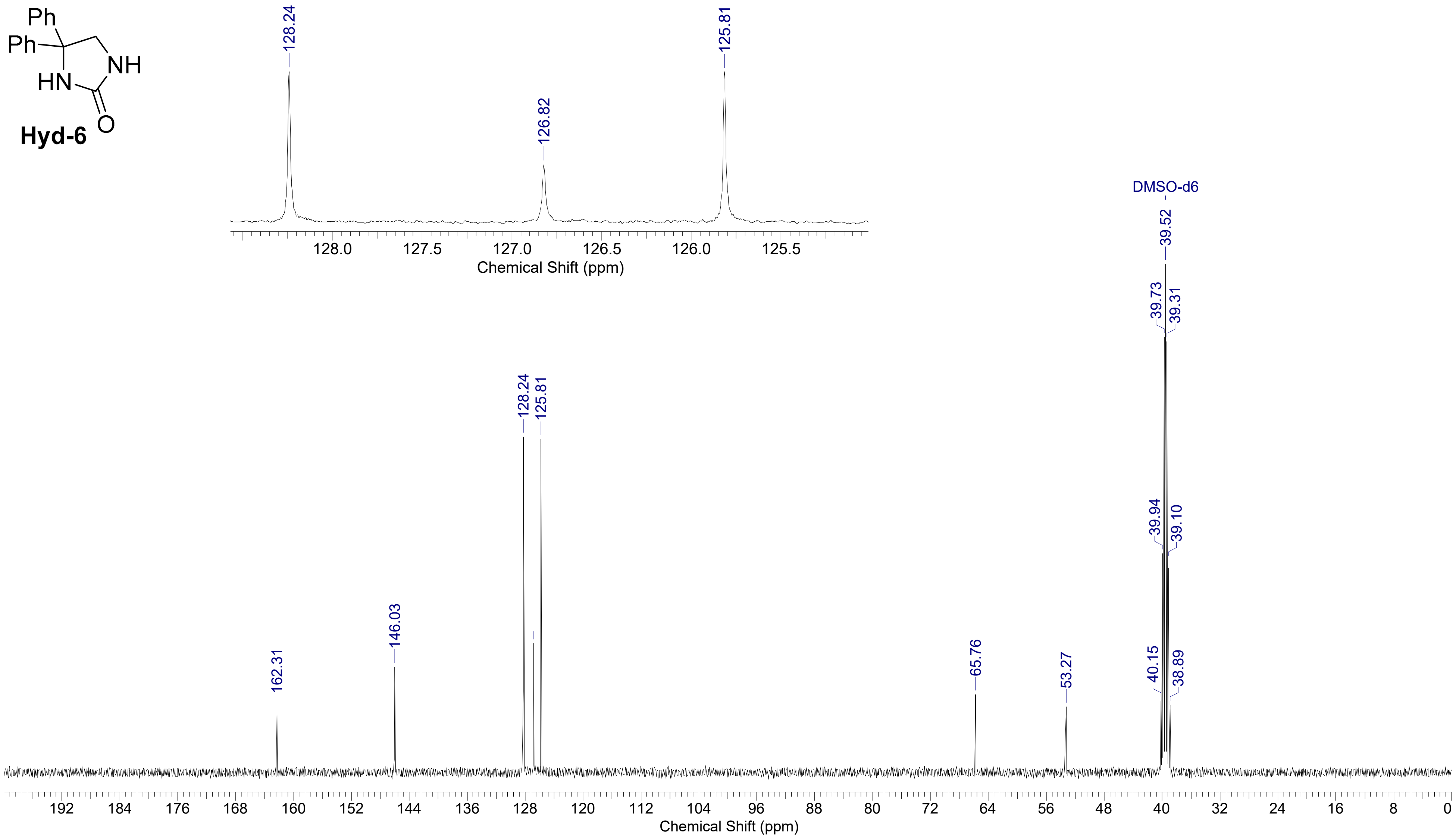

Figure S14. ^{13}C NMR (100.6 MHz, DMSO-d6) spectrum for **Hyd-6**

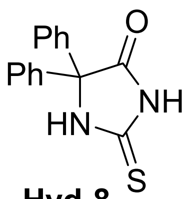

Hyd-8

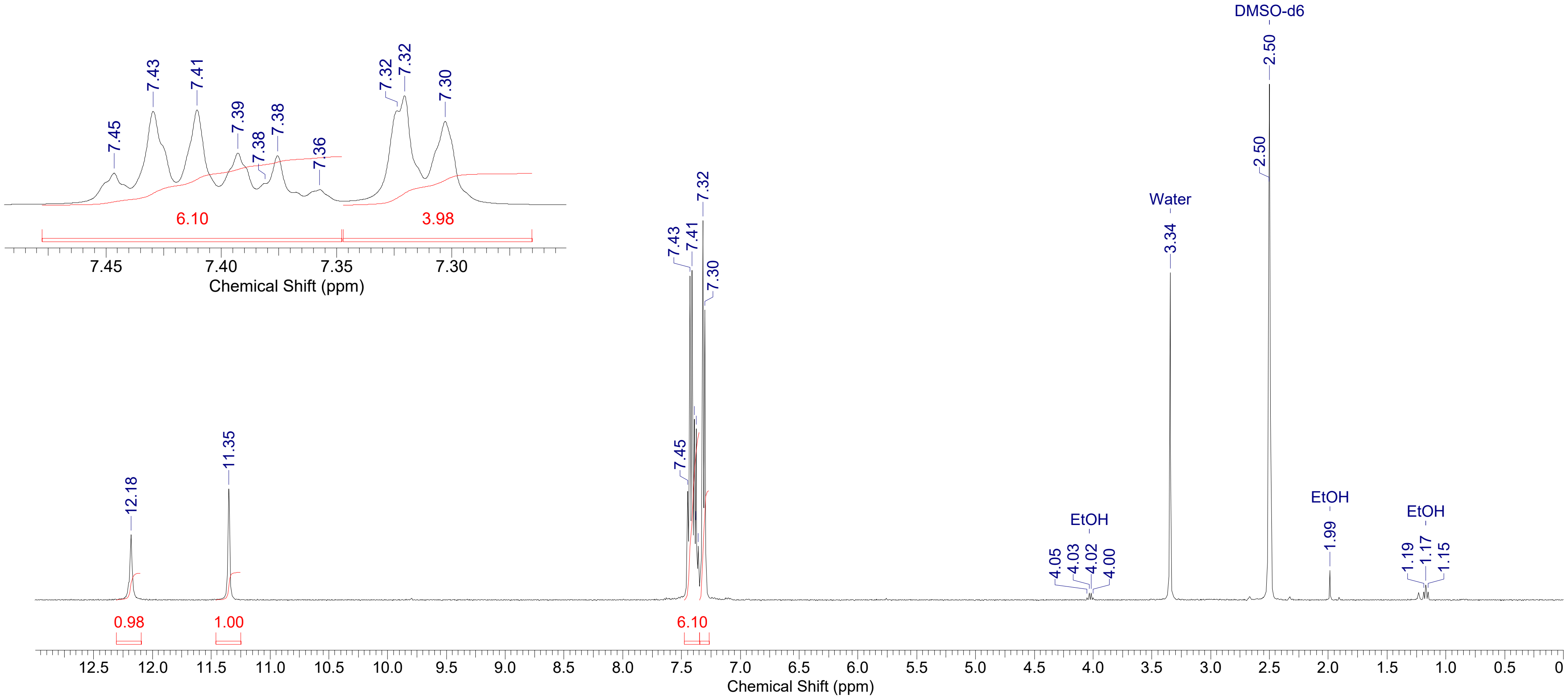

Figure S15. ^{1}H NMR (400 MHz, DMSO-d6) spectrum for **Hyd-8**

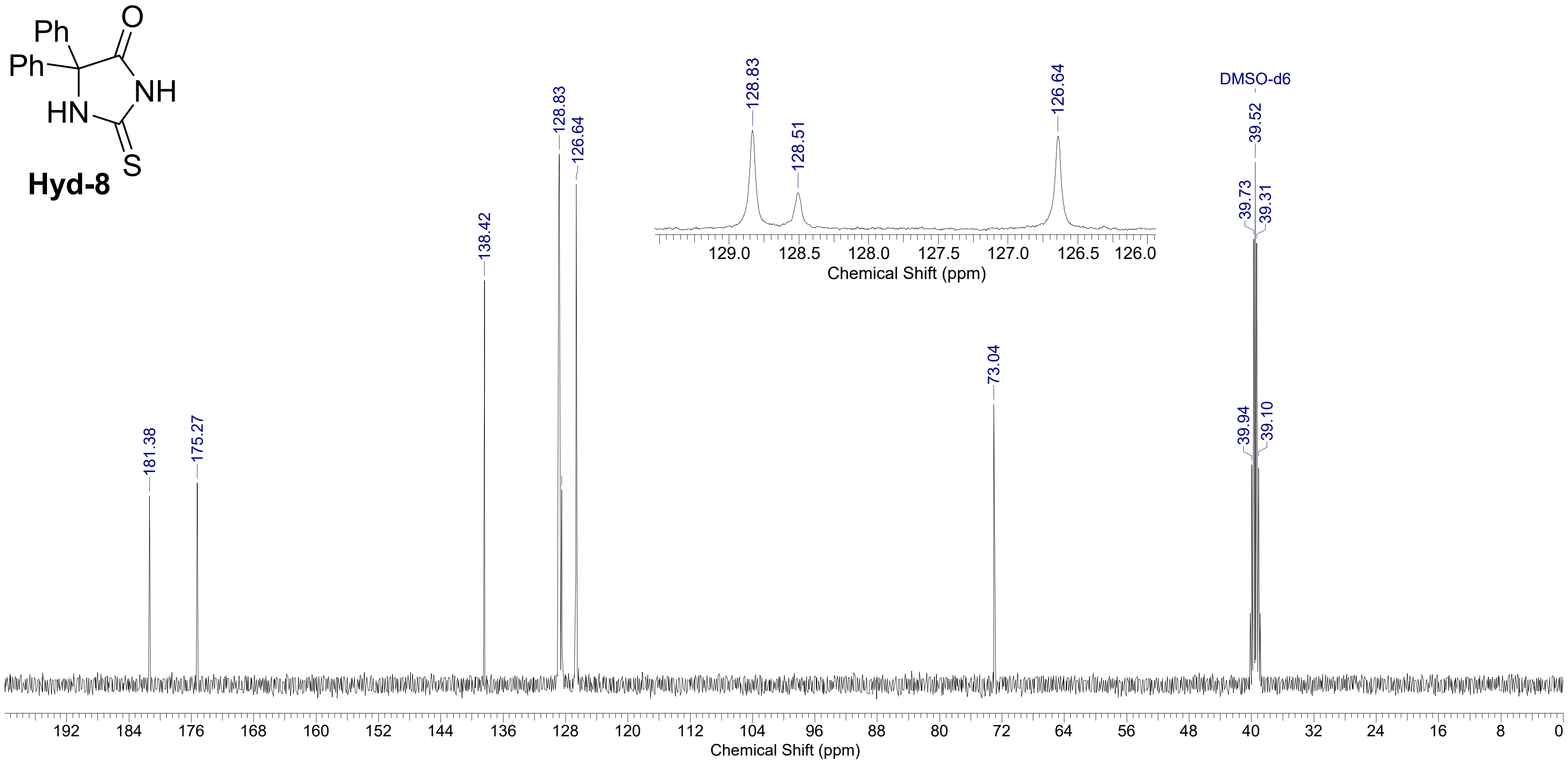

Figure S16. ^{13}C NMR (100.6 MHz, DMSO-d6) spectrum for **Hyd-8**

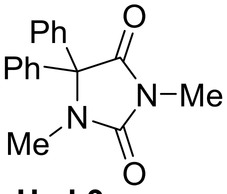

Hyd-9

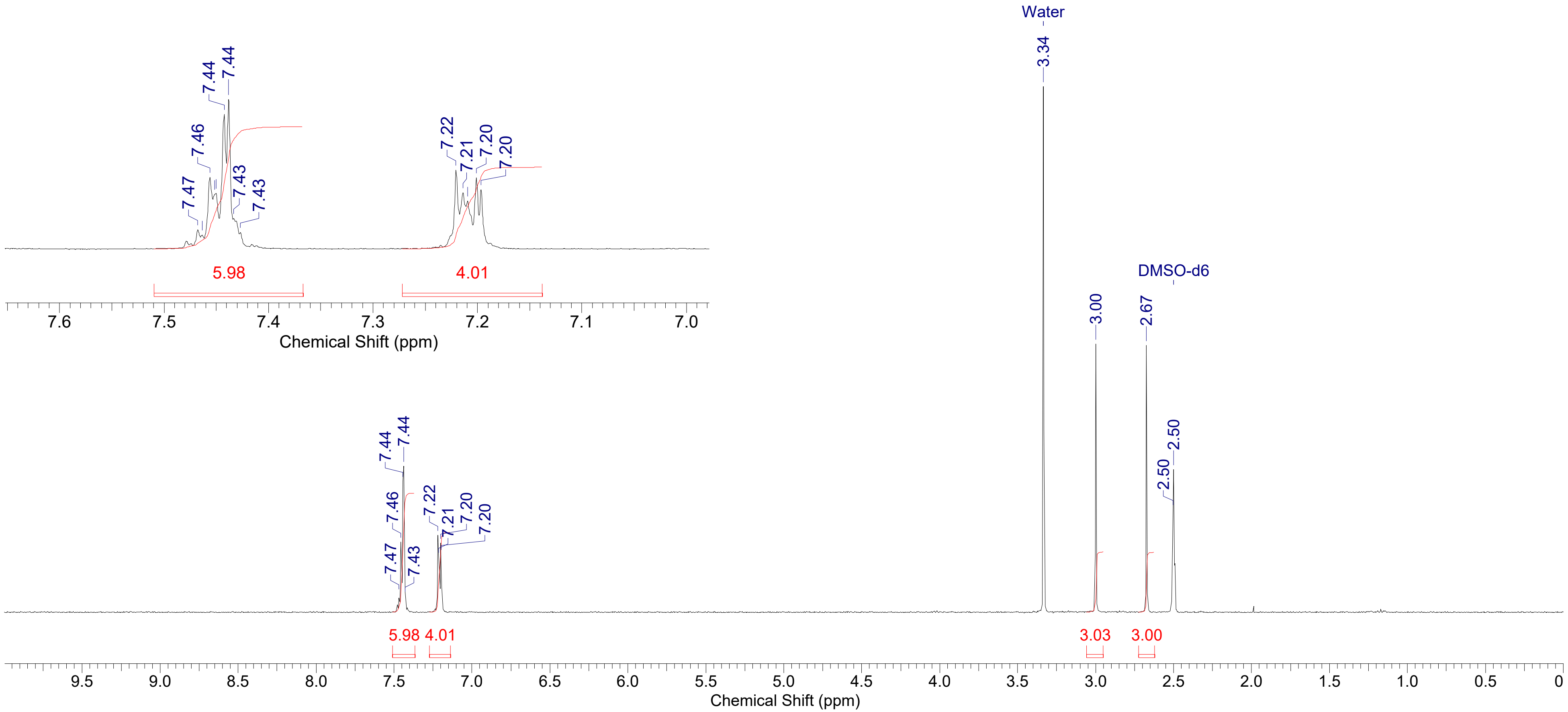

Figure S17. ^{1}H NMR (400 MHz, DMSO-d6) spectrum for **Hyd-9**

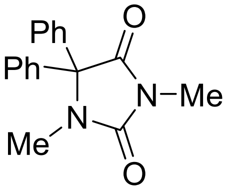

Hyd-9

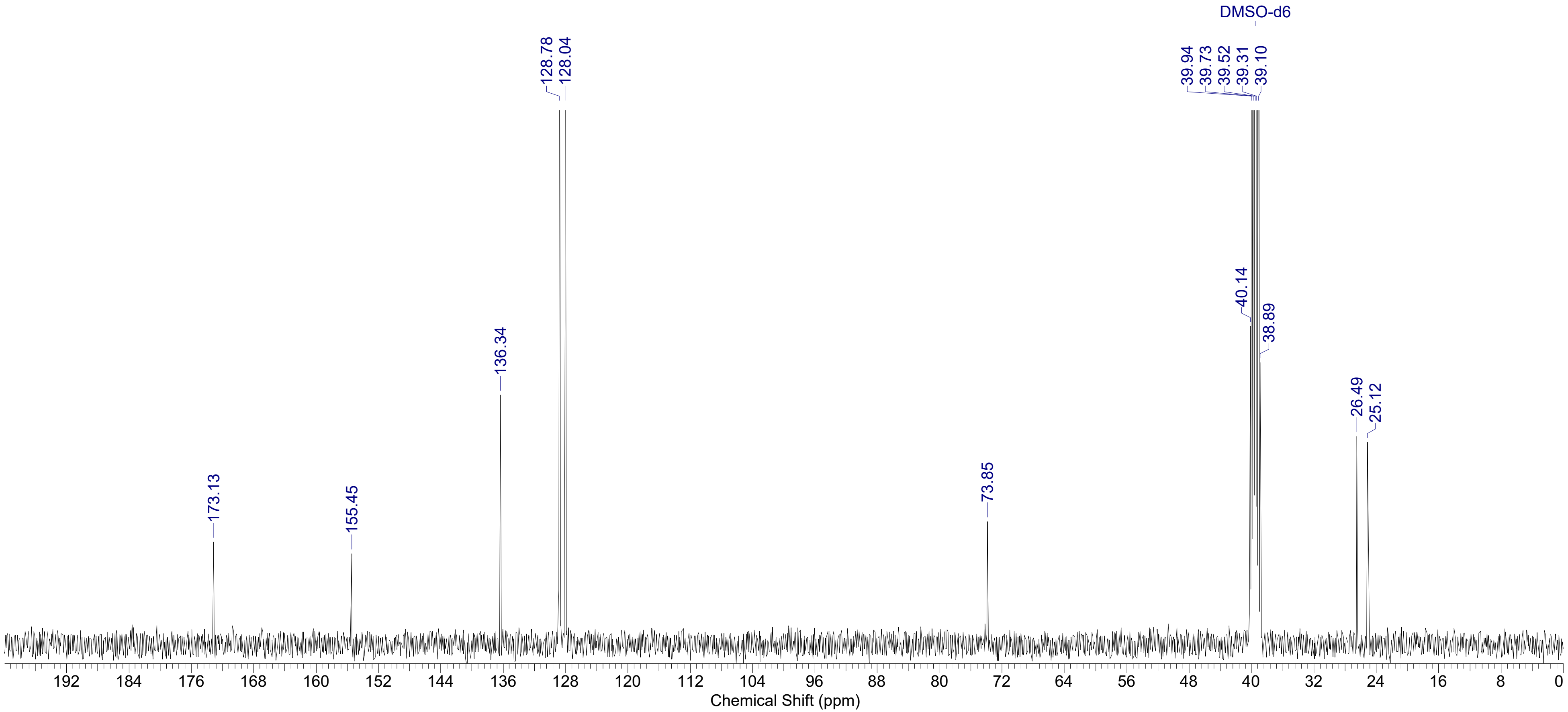

Figure S18. ^{13}C NMR (100.6 MHz, DMSO-d6) spectrum for **Hyd-9**

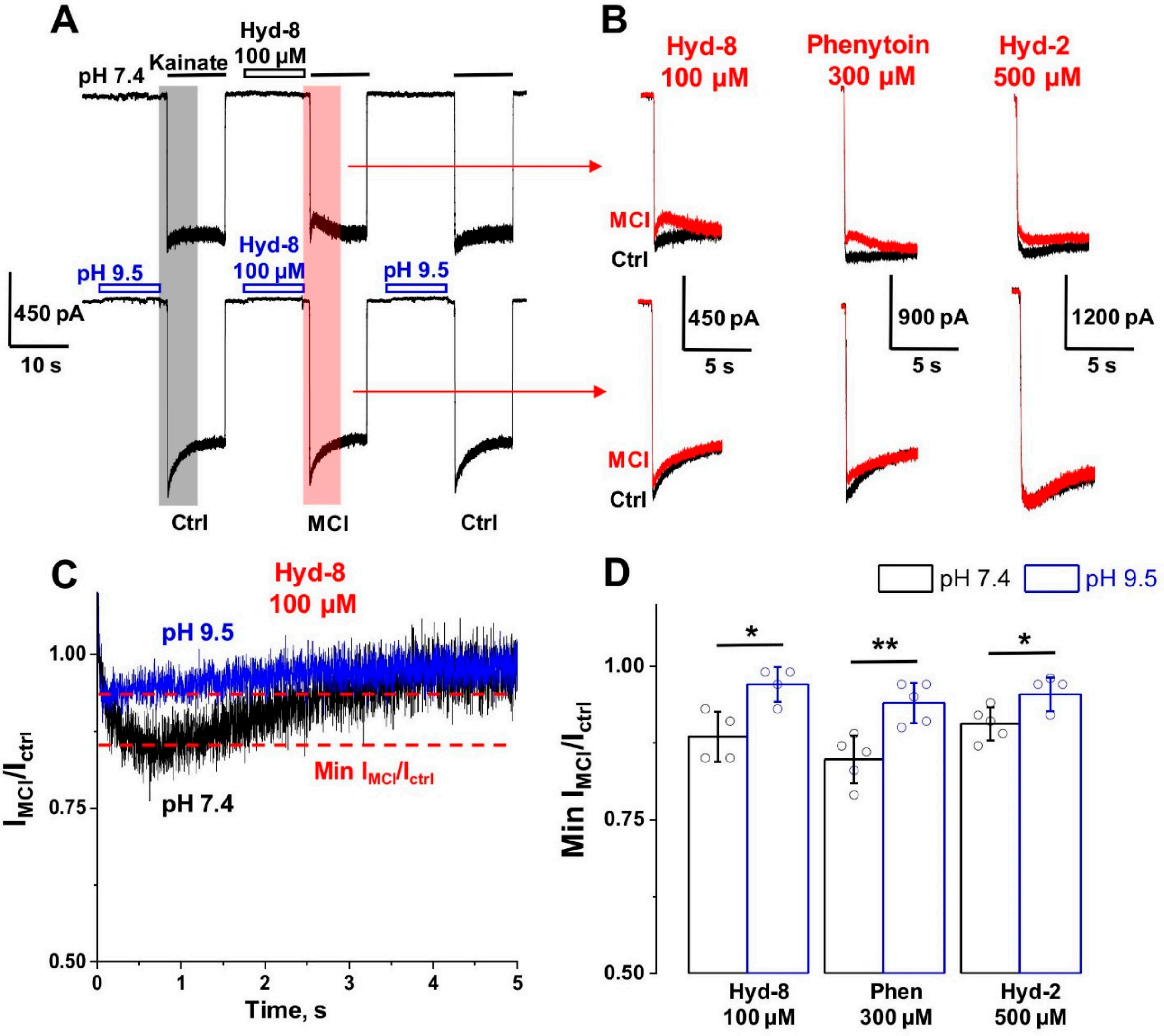

Figure S19. Membrane-to-channel inhibition (MCI) of CI-AMPARs by Hyd-8, phenytoin and Hyd-2. A. Protocol consisted of control kainate response, application of compound (Hyd-8 is shown) without kainate, 1 s pause in extracellular solution, testing application of kainate (MCI), and another application of kainate for washout indicating. Compounds were applied at pH 7.4 (above) or at pH 9.5 (below). In the latter case, additional application of solution with pH 9.5 was used before control responses for avoiding the effects related with pH-dependence of CI-AMPARs. B. Detailed representation of control and MCI responses for Hyd-8 (100 μM), phenytoin (300 μM) and Hyd-2 (500 μM) at pH 7.4 (above) and pH 9.5 (below). C. I_{MCI}/I_{Ctrl} ratio representation for first 5 s of kainate application for MCI at pH 7.4 (black trace) and at pH 9.5 (blue trace). Red dotted lines indicate minimal values (Min I_{MCI}/I_{Ctrl}), which were used for quantitative MCI assessment. D. Summary of MCI data for all compounds at pH 7.4 and 9.5. MCI is stronger at pH 7.4, suggesting that only uncharged molecules can reach their binding sites in the channel through a lipid layer. ***-p<0.05, **-p<0.01, paired t-test.**
